# Antiviral and Immunomodulatory Effects of 7-Deaza-2-methyladenosine (7DMA) in a Susceptible Mouse Model of Usutu Virus Infection

**DOI:** 10.3390/v17121639

**Published:** 2025-12-18

**Authors:** Rebeca P. F. Rocha, Marina A. Fontoura, Fabrício Naciuk, Leonardo C. Oliveira, Alice Nagai, Amanda Bellini Silva, Alexandre Borin, Jaqueline S. Felipe, Marjorie Bruder, Lais D. Coimbra, Rafael Elias Marques

**Affiliations:** 1 Brazilian Biosciences National Laboratory (LNBio), Brazilian Center for Research in Energy and Materials (CNPEM), Campinas, SP 13083-100, Brazil; 2 Department of Molecular and Cell Biology, University of California, Berkeley, CA 94720, USA; 3 Department of Genetics, Evolution, Microbiology and Immunology, Institute of Biology, University of Campinas (UNICAMP), Campinas, SP 13083-862, Brazil

**Keywords:** Usutu virus, 7DMA, antiviral, mouse model, immunopathogenesis

## Abstract

Usutu virus (USUV) is an emerging arbovirus recently associated with outbreaks in Western Europe. Although USUV is typically associated with asymptomatic or nonspecific febrile disease, the occurrence of severe neuroinvasive forms of disease has raised concern. There is currently no antiviral treatment available for USUV infection; therefore, we sought to investigate the protective effects of the nucleoside analogue 7DMA against USUV. Adding to 7DMA’s activity against USUV in vitro reported by us and others, we found that 7DMA inhibits USUV replication at multiple stages in mammalian cell lines Vero CCL81 and SH-SY5Y. In vivo testing of 7DMA using the susceptible IFNAR^-/-^ mouse model indicated that 7DMA treatment significantly reduced USUV viremia and viral load in tissues and prolonged mice survival. The characterization of the protective effects of 7DMA indicated that treatment also altered immunological aspects of disease development, further increasing the expression of mediators such as CXCL10, IL-15, and IFN-γ, and increasing neutrophil recruitment to target organs. We did not observe significant tissue damage or pathology in USUV-infected mouse brains, suggesting that systemic infection and disease are the major components leading to mortality in this model. We conclude that 7DMA exerts protective effects against USUV infection in the IFNAR^-/-^ model.

## 1. Introduction

The emergence and re-emergence of viral pathogens is an inevitable consequence of rapid urbanization, global trade, and climate change, all of which reshape the interactions between vectors, hosts, and ecosystems. Arboviruses are of particular concern, as their transmission dynamics are highly sensitive to environmental changes that influence mosquito distribution and vector competence [1,2]. Among them, Usutu virus (USUV), an encephalitic, mosquito-borne Orthoflavivirus within the Japanese encephalitis virus serocomplex, has gained increasing attention [3,4,5].

Facilitated by environmental change and the widespread distribution of Culex mosquito vectors, USUV has expanded beyond its original range and is now regularly detected across Europe [3,5]. USUV circulates predominantly in wild bird populations, particularly passerines, where it has been associated with encephalitis and significant mortality events, raising concerns for biodiversity and the potential for zoonotic spillover [6].

In humans, infections are often asymptomatic or mild, as observed with most Orthoflaviviruses, but can occasionally progress to severe neurological disease [7,8,9]. Adding to this concern, the virus shares close phylogenetic and ecological relationships with West Nile virus (WNV), which complicates diagnostics and frequently leads to underdiagnosis or misclassification in regions where both viruses co-circulate [9,10]. These factors collectively underscore the urgent need to advance our understanding of USUV epidemiology and pathogenesis, as well as to develop suitable animal models that can inform both preventive strategies and therapeutic interventions [11].

The nucleoside analog 7-deaza-2′-C-methyladenosine (7DMA) has broad-spectrum antiviral activity against several RNA viruses, including Zika virus and Hepatitis C virus [12,13]. Following intracellular phosphorylation to its active triphosphate form, 7DMA selectively targets viral RNA-dependent RNA polymerases, sparing host polymerases. The 2′-C-methyl modification interferes with RNA chain elongation, leading to non-obligate chain termination and inhibition of viral replication [12,14]. Although 7DMA is not approved for human use, this compound has been tested in preclinical models, particularly in the study of emerging and neglected viral infections for which no approved treatments currently exist [15]. In experimental settings, virus resistance to 7DMA requires extended exposure and serial passaging over at least 12 days [16]. In this scenario, 7DMA provides a valuable platform for dissecting the relationships between viral replication, host immune activation, and disease progression, becoming a valuable tool to explore the complex balance between antiviral immunity and immunopathology of acute infections, offering insights that may guide the rational design of next-generation antiviral therapies and host-directed interventions [15].

In this study, we aimed to evaluate the antiviral potential of 7DMA against USUV infection in mice. We used type I interferon receptor knockout (IFNAR^-/-^) mice, a well-established model for studying arboviral infections and evaluating antiviral efficacy in vivo, to observe that USUV rapidly reached the brain at early stages. As the disease progressed, in the absence of a type I interferon response, the viral load remained highest in the serum and brain but was also detected in the liver and spleen. Expression of proinflammatory cytokines such as CXCL1, CCL11, CXCL10, and G-CSF, and immune cell infiltration were predominantly observed in the spleen. In the brain, proinflammatory cytokines were elevated, but without evidence of immune cell infiltration or microglial activation despite the presence of the virus. Treatment with 7DMA delayed mortality and reduced viral loads in all organs. Moreover, treatment was associated with increased expression of CXCL10, IFN-γ, and IL-1β, and decreased levels of G-CSF, LIF, and CCL11, indicating that 7DMA influences the inflammatory profile across target organs. While these changes may result from reduced viral replication, they reflect a shift in host–pathogen dynamics during infection. Together, these findings underscore the critical role of type I interferon signaling in restricting USUV-induced disease. Importantly, we demonstrate that 7DMA reduces viral replication in vivo, and that its protective effect is accompanied by marked changes in the host inflammatory response.

## 2. Materials and Methods

### 2.1. Cell Lines and Viruses

Vero CCL81 (cat. 0245) and SH-SY5Y (cat. 0223) cell lines were purchased from the BCRJ cell bank (BCRJ; Rio de Janeiro, RJ, Brazil). Usutu virus (USUV; GenBank accession number KJ438705.1), isolate 12 1477, was provided by Dr. Schimidt-Chanasit from the Benhard-Nocht Institute of Tropical Medicine, Germany. Vero cells were cultured in Dulbecco’s modified Eagle medium (DMEM; Cultilab, Campinas, Brazil) supplemented with 10% of fetal bovine serum (FBS; Cultilab, Campinas, Brazil). SH-SY5Y cells were grown in 1:1 DMEM/F12 medium (Cultilab, Campinas, Brazil), supplemented with 1% of penicillin/streptomycin (Gibco, Thermo Fisher Scientific, Grand Island, NY, USA) and 10% FBS. Both cell types were maintained at 37 °C in 5% CO_2_ atmosphere.

### 2.2. Mice

Mice of the type I interferon receptor knockout strain (B6.129S2-Ifnar1tm1Agt/Mmjax; JAX #010830), here referred to as ‘IFNAR^-/-^ mice’, were housed in a pathogen-free ABSL-2 facility at the Brazilian Biosciences National Laboratory (LNBio/CNPEM, Campinas, Brazil). Animals were maintained under controlled environmental conditions (21–24 °C temperature; 12 h light/dark cycle) with unrestricted access to food (UV-irradiated; Nuvilab, Curitiba, Brazil) and water. All procedures were reviewed and approved by the Institutional Animal Care and Use Committee (CEUA/CNPEM, protocol #45) and conducted in accordance with institutional and national guidelines.

### 2.3. Usutu Virus Production and Stocks

USUV stocks were propagated in a monolayer of Vero CCL81 cells in T75 flasks (Sarstedt, Nümbrecht, Germany) using a single passage to minimize adaptation to cell culture [17]. At 96 h post-infection, cell supernatant was collected, clarified by centrifugation, aliquoted, and stored at −80 °C. USUV stocks were determined by virus plaque assay.

### 2.4. 7DMA Compound

The antiviral agent 7-deaza-2′-methyladenosine (7DMA) was synthesized in-house by the Medicinal Chemistry group at LNBio/CNPEM using a three-step procedure as previously described by Naciuk et al., 2023 [18]. 7DMA was resuspended in DMSO and subsequently diluted in vehicle at experiment-specific concentrations in both in vitro and in vivo assays, with the final DMSO concentration at 0.001% in culture medium and drinking water, respectively.

### 2.5. Virus Plaque Assay

USUV viral load in samples was quantified using a virus plaque-forming assay in Vero cell monolayers in 24-well plates. Supernatants were clarified before use, and mouse tissue samples were homogenized in DMEM at 10% (*w*/*v*). A six-point 10-fold serial dilution series was prepared for each sample, and the dilutions were applied to Vero monolayers. After 1 h of adsorption at 37 °C in 5% CO_2_, the inoculum was removed and replaced with a semi-solid overlay consisting of DMEM supplemented with 1.5% carboxymethylcellulose (CMC; Synth, São Paulo, Brazil) and 5% FBS Seven days post-infection, cells were fixed with 8% paraformaldehyde (PFA; Sigma, St. Louis, MO, USA) and stained with 1% methylene blue (Sigma, USA). Viral titers were expressed as plaque-forming units (PFU) per milliliter of supernatant or per 100 mg of tissue, with a limit of detection of 40 PFU/mL or 40 PFU/100 mg.

### 2.6. In Vitro Dose Response Assay

Vero CCL81 or SH-SY5Y cells were seeded in 24-well plates at a density of 5 × 10^5^ cells per well. After 24 h, monolayers were infected with USUV at a multiplicity of infection (MOI) of 0.1. Following 1 h of viral adsorption, the inoculum was removed, and cells were treated with vehicle (0.001% DMSO) or 7DMA at varying concentrations in the culture medium. Cell supernatants were collected at 24 and 48 h post-infection for viral titration by plaque assay.

### 2.7. Time of Addition Assay

Vero CCL81 or SH-SY5Y cells were seeded in 24-well plates at a density of 5 × 10^5^ cells per well and, after 24 h, infected with USUV at a multiplicity of infection (MOI) of 0.1. Following 1 h of viral adsorption, the inoculum was removed, and 7DMA was added at 50 μM to the well corresponding to 0 h. Subsequently, at 2 h intervals, individual wells were treated once with the compound until 24 h post-infection (h.p.i.), when supernatants were collected for viral titration by plaque assay.

### 2.8. In Vivo Experimental Infection and Survival Assay

IFNAR^-/-^ mice, aged 8–12 weeks, were infected with 10^4^ PFU of USUV (100 μL) or phosphate-buffered saline (mock) through the subcutaneous route. Animals were treated by gavage daily with 7DMA (50 mg/kg) or vehicle (0.001% DMSO in drinking water) from the day of infection through day 6. The selected dose was based on previously reported safe and effective levels in the literature [12,19,20]. For survival experiments, mice were observed daily for 14 days. Sample collection time points were established at day 3 post-infection and the peak of disease onset (day 4 or 5 p.i.). Mice suffering from severe pain were euthanized according to the humane endpoint established for this study and counted dead for the survival analyses.

### 2.9. Hematological Analysis

Mice were anesthetized prior to blood collection. Blood was obtained by incision of the brachial plexus and collected with a Pasteur pipette containing 0.1% EDTA, then transferred to EDTA-coated tubes. Total leukocyte counts were determined using a Neubauer chamber after a 1:10 dilution of blood in Türk’s solution. Cell counts were corrected according to the dilution factor and the Neubauer chamber correction factor (10^4^). For differential leukocyte counts, blood smears were prepared and stained using a panoptic staining kit, followed by microscopic analysis. Data are expressed as the percentage of each leukocyte subtype relative to the total leukocyte population.

### 2.10. Multiplex ELISA

Cytokine and chemokine levels in mouse spleen, liver, brain, and serum were quantified using the MILLIPLEX MCYTOMAG-70K multiplex assay (Millipore, Billerica, MA, USA). Tissue samples (spleen, liver, and brain) were processed at a 10% (*w*/*v*) ratio in cytokine extraction buffer (0.05% Tween, 0.5% BSA, 0.01 M phenylmethylsulfonyl fluoride [PMSF], 0.1 M benzethonium chloride, 0.1 M EDTA, and 1 μM aprotinin in 0.1 M phosphate-buffered saline). Multiplex assays were performed according to the manufacturer’s instructions at the Life Sciences Core Facility (LaCTAD, UNICAMP, Campinas, Brazil). Results are expressed as picograms per 100 mg of tissue or per milliliter of serum.

### 2.11. Flow Cytometry

At 4 days post-infection (d.p.i.), mice were anesthetized and perfused with 20 mL of 0.1 M PBS prior to brain collection. Brains were pooled in pairs and homogenized in RPMI medium using a Dounce homogenizer. Samples were digested for 30 min at 37 °C with 20 μg/mL collagenase and 10 μg/mL DNase. Leukocytes were isolated by filtration through a 100-μm cell strainer (Greiner Bio-One, São Paulo, SP, Brazil) followed by separation on a 70%:30% Percoll gradient (Sigma-Aldrich, St. Louis, MO, USA). Cells were washed with PBS, resuspended, and counted. For staining, 10^6^ cells were seeded per well in 96-well V-bottom plates. Cells were stained with FVS575 viability dye and subsequently blocked with 0.5 μg/mL Fc-block (BioLegend, San Diego, CA, USA). Next, cells were incubated with two distinct antibody panels, each prepared in staining buffer (BD Biosciences, Franklin Lakes, NJ, USA), for lymphocyte and phagocyte immunophenotyping (markers listed in Table 1). Compensation beads (BD Biosciences, #552845 and #552843) were stained with each antibody for compensation controls. Following staining, cells were washed in staining buffer and fixed in 2% PFA/PBS. Cells were analyzed using BD FACSymphony A5 flow cytometer from the Immunology Department at the Institute of Biology/UNICAMP.

For lymphocyte analysis, cells with low side scatter (SSC) were gated, followed by doublet exclusion using FSC-A × FSC-H parameters. Live cells were identified by FVS575– gating. Leukocytes were defined as CD45^+^, from which CD4^+^ cells were classified as CD4^+^ T lymphocytes and CD8^+^ cells as CD8^+^ T lymphocytes. Myeloid cell analysis was initiated using a broader SSC-A × FSC-A gate, with singlets and live leukocytes selected as described above. Dendritic cells were defined as CD11c^+^, while neutrophils were identified as CD11b^+^Ly6G^+^. Within the Ly6G^+^ population, CD44 expression was further evaluated in blood and spleen samples. Ly6G^−^ cells were analyzed for F4/80 expression, and F4/80^+^ cells were classified as macrophages. The complete gating strategies for spleen, blood, and brain are shown in the Supplementary Materials, Figures S3–S5, respectively.

### 2.12. Histological and Immunostaining Analysis

Mouse brains were collected and rinsed in 0.1 M PBS, then fixed in 4% paraformaldehyde (PFA) in PBS at 4 °C for 48 h. Samples were processed through graded ethanol and xylene, embedded in paraffin (Paraplast Plus, Sigma-Aldrich, St. Louis, MO, USA), and sectioned coronally at 6 μm with an RM2255 microtome (Leica, Buffalo Grove, IL, USA). Coronal slices were stained with hematoxylin and eosin (H&E) and analyzed using FS DM6 microscope (Leica) at ×20 magnification. For immunofluorescence, paraffin-embedded slices were dewaxed, rehydrated, and subjected to antigen retrieval in Tris-EDTA buffer (pH 9) at 86 °C for 30 min. After permeabilization and 1% BSA blocking, slices were incubated overnight at 4 °C with anti-IBA1 antibody (1:600; Novus Biologicals, Centennial, CO, USA), followed by Alexa Fluor 647-conjugated anti-rabbit secondary antibody (1:800; Abcam, Cambridge, UK) for 2 h at room temperature. Nuclei were counterstained with DAPI and slides were mounted with Dako Shield (Dako, Glostrup, Denmark) for analysis on a TCS SP8 confocal microscope (Leica) at ×40 magnification.

### 2.13. Statistical Analysis

Statistical analyses were performed as described in Figure legends. Comparisons between groups were performed by first analyzing infected versus mock samples to assess the effect of infection, followed by comparisons between treated and vehicle-treated samples within the infected group to evaluate treatment effects. Descriptive statistics of the distribution of multiplex ELISA data were also performed to determine mean, minimum, maximum and standard deviation (SD), available in Supplementary Table S1. *p*-values under 0.05 were considered significant in all cases. Curve comparison and statistical analysis were performed in GraphPad Prism software version 8 (GraphPad Software, La Jolla, CA, USA).

## 3. Results

### 3.1. 7DMA Inhibits USUV Replication in Mammalian Cell Lines at Multiple Stages

**Figure 1:**
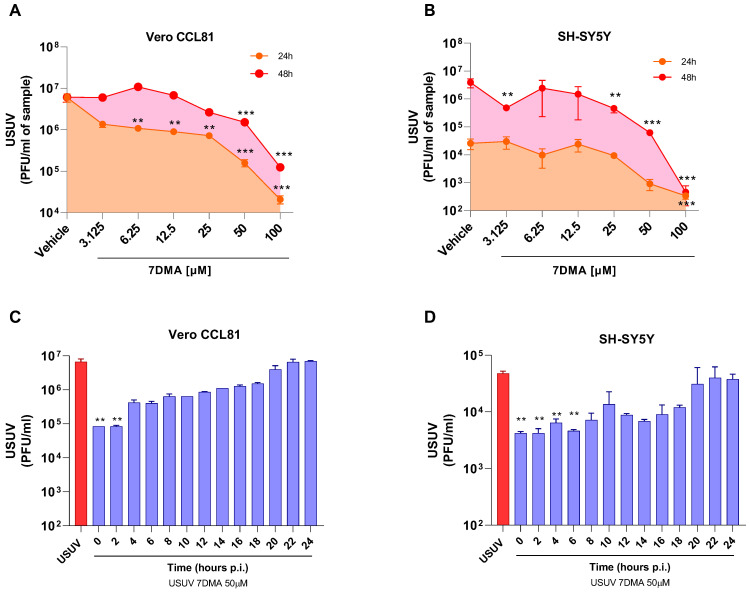
7DMA inhibits USUV replication in mammalian cell lines. Dose-dependent inhibition of USUV replication by 7DMA in (**A**) Vero CCL81 and (**B**) SH-SY5Y cells. Cells were infected and treated with vehicle or 7DMA in concentrations ranging from 3.1 to 100 μM. Cell culture supernatant was collected at 24 or 48 h post-infection for the assessment of infectious viral load. Time-of-addition assay of 7DMA at 50 μM in (**C**) Vero CCL81 or (**D**) SH-SY5Y cell culture infected with USUV. was collected at 24 h post-infection for the assessment of infectious viral load. USUV treated with vehicle (red bars); USUV treated with 7DMA (blue bars). Supernatant The right Y axis represents viral load results expressed in plaque-forming units per mL (PFU/mL) of culture supernatant. USUV replication data was analyzed using one-way analysis of variance with the non-parametric Kruskal–Wallis test. ** *p* < 0.01 and *** *p* < 0.001.

We and others have previously shown that 7DMA has dose-dependent antiviral activity against USUV infection in cell culture, and that concentrations up to 100 µM are not cytotoxic in either Vero or SH-SY5Y cells [18,21]. Further testing of 7DMA antiviral activity against USUV infection in Vero CCL81 and SH-SY5Y mammalian cell lines indicated that 7DMA treatment reduces USUV in cell culture supernatants at different times post-infection (p.i.). Vero CCL81 and SH-SY5Y cells were infected with USUV and treated or not with 7DMA (Figure 1A,B). Assessment of viral load in Vero CCL81 cell culture supernatants at 24 h and 48 h p.i. indicated that the antiviral effect of 7DMA is more pronounced in samples collected 24 h p.i., as lower concentrations (3125 to 25 µM) of 7DMA resulted in an approx. 10-fold reduction in viral load, while the same 7DMA concentrations had no effect on samples collected at 48 h p.i. (Figure 1A). Conversely, the assessment of USUV load in SH-SY5Y cell culture supernatants indicated that 7DMA had the same effect on samples collected at 24 or 48 h p.i. Importantly, 7DMA treatment presented an antiviral effect in both cell lines at the higher concentrations tested (50 and 100 µM), resulting in 10 to 100-fold reductions in viral load.

Considering that the antiviral effect of 7DMA in Vero CCL81 and SH-SY5Y is not identical, we performed a time-of-addition experiment with both cell lines to identify in which stages of the USUV replication cycle 7DMA might be acting (Figure 1C,D). Nucleoside analogues typically affect viral RNA replication, targeting the viral RNA-dependent RNA polymerase (RDRP) [22]. Our results showed, for both cell lines, that 7DMA treatment is most effective when added up to 6 h p.i. 7DMA also presents USUV inhibitory activity up to 18 h p.i., but from 20 h p.i. and on, at the late stages of USUV replication, addition of 7DMA does not reduce viral load, which was similar to the vehicle-treated control.

### 3.2. 7DMA Delays Mortality and Reduces Viral Load in a Susceptible Mouse Model of USUV Infection

**Figure 2:**
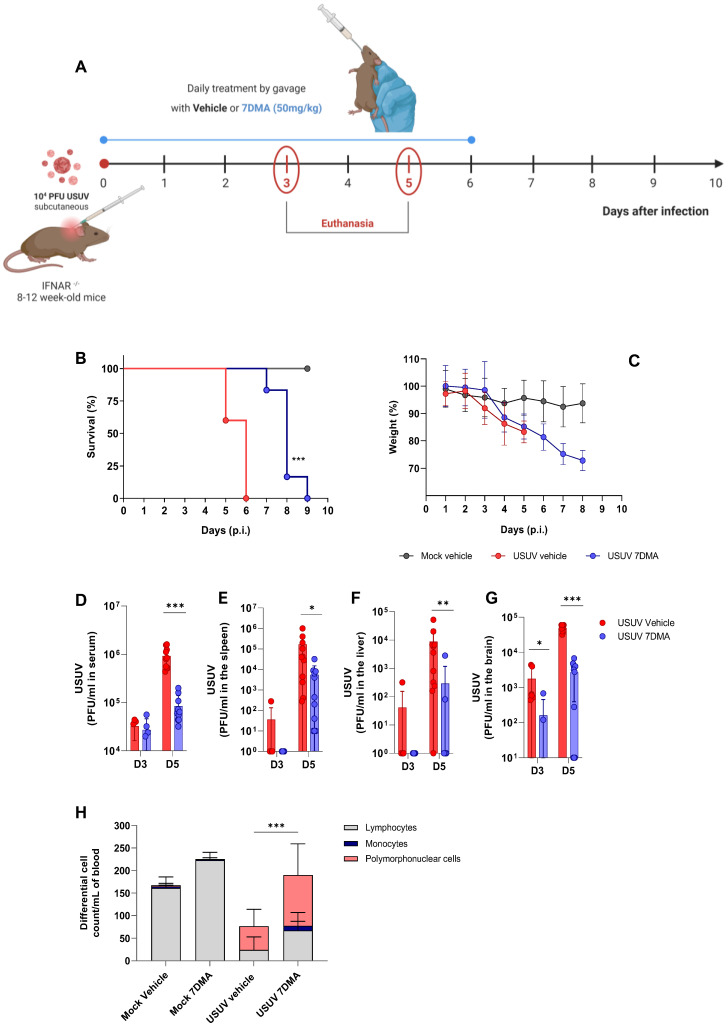
7DMA delays USUV-induced disease and mortality in IFNAR^-/-^ mice. (**A**) Schematic representation of experimental design. Adult IFNAR^-/-^ mice were infected with 10^4^ PFU of USUV subcutaneously and treated daily until 6 days post-infection with 7DMA (50 mg/mg) (blue) or vehicle (red). Survival (**B**) and weight loss (**C**) analyses. 7DMA treatment reduces USUV viral load in (**D**) serum, (**E**) spleen, (**F**) liver, and (**G**) brain of adult IFNAR^-/-^ mice. (**H**) Treatment with 7DMA recovered the total leukocyte count in blood, with a significant increase in polymorphonuclear cells. Data from two independent experiments, conducted with at least 5 mice per group. Survival curves were compared using the Log-Rank test for trend. Viral plaque assays were analyzed using the Mann–Whitney U test. Statistical analysis of total and differential leukocyte counts was performed using two-way ANOVA followed by Tukey’s multiple comparison test. * *p* < 0.05; ** *p* < 0.01 and *** *p* < 0.001.

We progressed to the testing of 7DMA against USUV infection in vivo, using the susceptible IFNAR^-/-^ mouse model [23] (Figure 2). Adult mice were inoculated with USUV using the subcutaneous route and treated or not with 7DMA orally, from the day of infection up to day 6 p.i. (Figure 2A). USUV-infected IFNAR^-/-^ mice receiving vehicle all died up to day 6 p.i., while IFNAR^-/-^ mice receiving 7DMA (50 mg/kg) survived up to day 9 p.i. (Figure 2B). On average, 7DMA treatment extended infected mice’s survival by approximately 50% (6 to 9 days), with the first deceased individuals detected only after 7DMA treatment was finished. Weight assessment during infection indicated that both 7DMA-treated and vehicle-treated mice developed disease, shown by a constant decrease in weight after day 3 p.i., in comparison to the non-infected control (Figure 2C).

In parallel, groups of mice were infected with USUV and treated or not with 7DMA and euthanized at days 3 and 5 p.i., preceding the onset of death in the USUV-infected vehicle-treated group, for sample collection. The assessment of infectious viral load in USUV-infected IFNAR^-/-^ mouse tissues indicated that USUV was detectable in serum and all organs evaluated at day 3 p.i., with the largest viral load found in the mouse brain. 7DMA treatment significantly reduced viral load in the brain at day 3 p.i., in comparison to the vehicle-treated control (Figure 2D–G). At day 5 p.i., preceding the USUV-induced death in this model, viremia and viral load in the spleen, liver, and brain were elevated in comparison to day 3 p.i. 7DMA treatment resulted in significant reductions in viral load in all evaluated tissues in comparison to the vehicle-treated control (Figure 2D–G). Importantly, 7DMA led to undetectable viral loads in some tissue samples, which were assessed using a virus plaque-forming assay for the quantification of infectious USUV. Also, leukocyte counts in peripheral blood indicated that USUV-infected IFNAR^-/-^ mice, treated or not with 7DMA, developed lymphopenia after infection (Figure 2H). Conversely, both groups of infected mice also developed neutrophilia in response to USUV infection. In 7DMA-treated mice, increases in polymorphonuclear cells and monocytes largely compensated for the reduced number of circulating lymphocytes, with total counts that were similar to mock-infected controls. Vehicle-treated or 7DMA-treated mock-infected IFNAR^-/-^ mice presented normal numbers of circulating leukocytes, although 7DMA-treated mice presented a greater number of circulating lymphocytes on average.

In summary, we observed that 7DMA treatment in vivo resulted in delayed USUV-induced mortality, which was associated with significantly reduced viral loads in mouse tissues and alterations in the distribution of circulating leukocytes. While these immune changes coincided with treatment, we cannot determine whether they result directly from 7DMA or reflect a secondary effect due to reduced viral replication.

### 3.3. 7DMA Treatment Alters the Cytokine and Leukocyte Responses to USUV Infection in the IFNAR^-/-^ Mice

We sought to characterize USUV-induced disease progression with or without 7DMA treatment in the IFNAR^-/-^ mice. As described above, groups of mice were infected with USUV and treated or not with 7DMA and euthanized at day 5 p.i. for the collection of whole blood, spleen, liver, and brain samples (Figure 2A). Samples were processed and analyzed using a multiplexed ELISA assay for the quantification of 23 different cytokines and chemokines, or using flow cytometry, to characterize leukocyte populations recruited and activated in each tissue.

Multiplex ELISA results showed that USUV infection induces the expression of multiple cytokines and chemokines in different tissues: G-CSF, GM-CSF, IL-1α, IL-1β, IL-6, IL-15, LIF, IFN-γ, CXCL1, CXCL5, CXCL10, and CCL11, either at day 3 or day 5 p.i. (Supplemental Figure S1 and Figure 2). Statistical analysis of cytokine/chemokine expression induced by USUV in the mouse spleen, liver, and brain in comparison to mock-infected controls indicates that most mediators were increased in both 7DMA-treated and nontreated mice (Supplementary Table S1). However, 7DMA treatment led to changes in cytokine and chemokine levels in the infected tissue, with some being reduced and others elevated in response to USUV infection.

**Figure 3:**
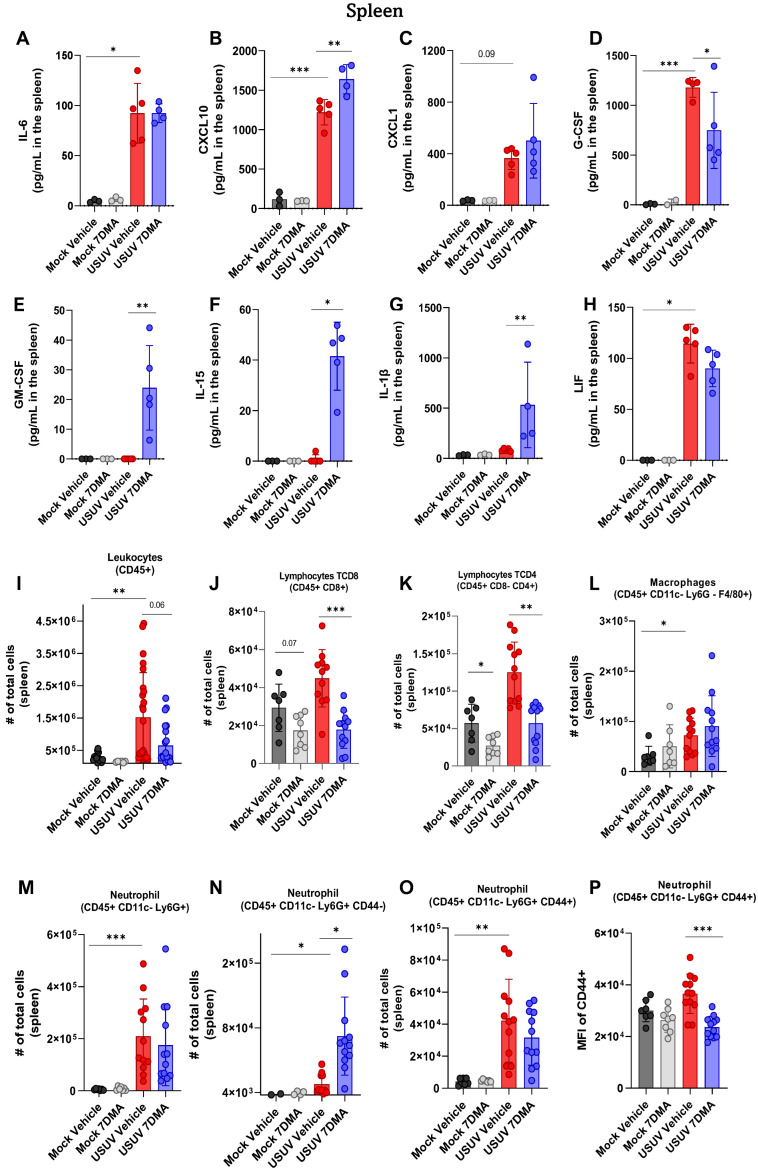
7DMA alters the splenic inflammatory response to USUV infection in IFNAR^-/-^ mice, affecting the expression of key immune mediators. USUV infection increases the levels of (**A**) IL-6, (**B**) CXCL10, (**C**) CXCL1, (**D**) G-CSF, and (**H**) LIF in IFNAR^-/-^ spleen. 7DMA induces splenic expression of (**E**) GM-CSF, (**F**) IL-15, and (**G**) IL-1b in USUV-infected mice. Flow cytometry analysis shows that (**I**) leukocyte number is increased by USUV infection and reduced by 7DMA treatment, reflected by (**J**) CD8^+^ lymphocyte, (**K**) CD4^+^ lymphocyte, (**L**) macrophage, and (**M**–**P**) neutrophil response. Multiplex ELISA of 3 mice per Mock group and 5 mice per infected group. Cytokine and chemokine quantification data were analyzed using two-way ANOVA with Dunnett’s multiple comparison test. Flow cytometry experiment conducted with 8 mice per Mock group and 12 mice per USUV-infected group. Significance was determined by the Kruskal–Wallis test, followed by Dunn’s multiple-comparison test. * *p* < 0.05; ** *p* < 0.01 and *** *p* < 0.001.

Analysis of cytokines and chemokines expressed in the spleen of IFNAR^-/-^ mice indicated that USUV induced the expression of IL-6 (Figure 3A), CXCL10 (Figure 3B), CXCL1 (Figure 3C), G-CSF (Figure 3D), and LIF (Figure 3H), of which CXCL10 and G-CSF were expressed at the highest levels. Infected mice treated with 7DMA showed increased expression of CXCL10 and reduced expression of G-CSF and LIF. Importantly, 7DMA treatment also led to the expression of GM-CSF (Figure 3E), IL-15 (Figure 3F), and IL-1β (Figure 3G) in USUV-infected mice.

Analysis of leukocyte populations present in the spleen indicated that USUV infection caused an increase in the total number of leukocytes (Figure 3I). USUV-infected mice receiving 7DMA presented a smaller increase in the total leukocyte numbers in the spleen. A detailed analysis of leukocyte populations participating in this process showed an increased number of T lymphocytes, macrophages, and notably, neutrophils in USUV-infected spleens (Figure 3J–M). While 7DMA treatment in USUV-infected mice led to reduced numbers of T CD4 and T CD8 lymphocytes in comparison to infected vehicle-treated mice (Figure 3J,K), corroborating a reduced number of total leukocytes found in the 7DMA-treated group, the numbers of neutrophils and macrophages were not significantly affected by 7DMA treatment (Figure 3L,M). However, we noticed that 7DMA treatment promoted a significant increase in the number of CD44^-^ neutrophils (Figure 3N), while the number of CD44^+^ neutrophils remained similar between vehicle-treated and 7DMA-treated mice infected with USUV (Figure 3O). Moreover, the CD44^+^ neutrophil population in 7DMA-treated mice expressed lower levels of CD44 in comparison to vehicle-treated mice (Figure 3P), further showing that 7DMA treatment caused a significant shift in the neutrophil population recruited to the spleen in infected mice.

**Figure 4:**
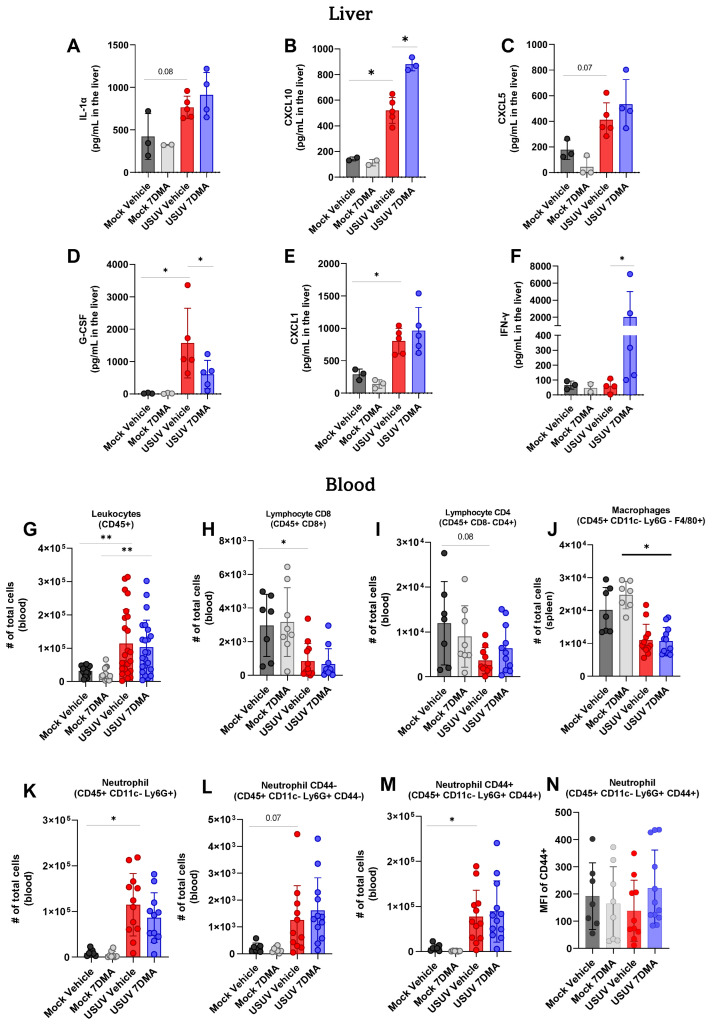
7DMA treatment impacts systemic response to USUV infection in mice. (**A**–**F**) USUV infection and 7DMA treatment alters the levels of IFN-γ cytokines/chemokines in IFNAR^-/-^ liver. USUV infection increases the number of (**G**) circulating CD45^+^ cells in IFNAR^-/-^ mouse blood. Although (**H**,**I**) lymphocyte and (**J**) macrophage populations showed reduction, (**K**–**N**) neutrophil populations presented higher numbers in infected blood. Multiplex ELISA of 3 mice per Mock group and 5 mice per infected group. Cytokine and chemokine quantification data were analyzed using two-way ANOVA with Dunnett’s multiple comparison test. Flow cytometry experiment conducted with 8 mice per Mock group and 12 mice per USUV-infected group. Significance was determined by Kruskal–Wallis test, followed by Dunn’s multiple-comparison test. * *p* < 0.05 and ** *p* < 0.01.

Analysis of cytokines and chemokines expressed in the liver of IFNAR^-/-^ mice indicated that USUV induced the expression of IL-1α (Figure 4A), CXCL10 (Figure 4B), CXCL5 (Figure 4C), G-CSF (Figure 4D), and CXCL1 (Figure 4E), of which G-CSF and CXCL1 were expressed at the highest levels. 7DMA treatment led to the increased expression of CXCL10 and, conversely, to a reduced expression of G-CSF, in USUV-infected mice. Importantly, 7DMA treatment also led to a significant increase in the expression of IFN-γ (Figure 4F) only in USUV-infected animals.

Finally, leukocyte populations circulating in peripheral blood were also analyzed using flow cytometry, and showed that USUV infection caused an increase in the total number of CD45^+^ leukocytes (Figure 4G) in mice receiving either vehicle or 7DMA. Conversely, circulating T CD8^+^ lymphocytes (Figure 4H) and macrophages (Figure 4J) were reduced in USUV-infected mice in comparison to uninfected controls in both vehicle-treated and 7DMA-treated mice. The number of circulating T CD4^+^ lymphocytes was also reduced in USUV-infected mice, though differences were not significant (Figure 4I). Similar to the spleen, USUV infection led to an increase in the number of neutrophils in the blood (Figure 4K–M), which was observed in both CD44^-^ and CD44^+^ populations, with CD44^+^ neutrophils representing the largest neutrophil population in the blood. Overall, in comparison to infected vehicle-treated mice, 7DMA treatment did not alter any leukocyte population evaluated.

Overall, the analysis of cytokines/chemokines induced by USUV infection, and the leukocyte populations involved, indicated that USUV infection leads to a robust and systemic inflammatory response. 7DMA treatment affects this inflammatory response, leading to changed levels of inflammatory mediators and leukocyte recruitment, notably biased towards neutrophils in the spleen.

### 3.4. 7DMA Alters Cytokine and Chemokine Expression in USUV-Infected Brains

**Figure 5:**
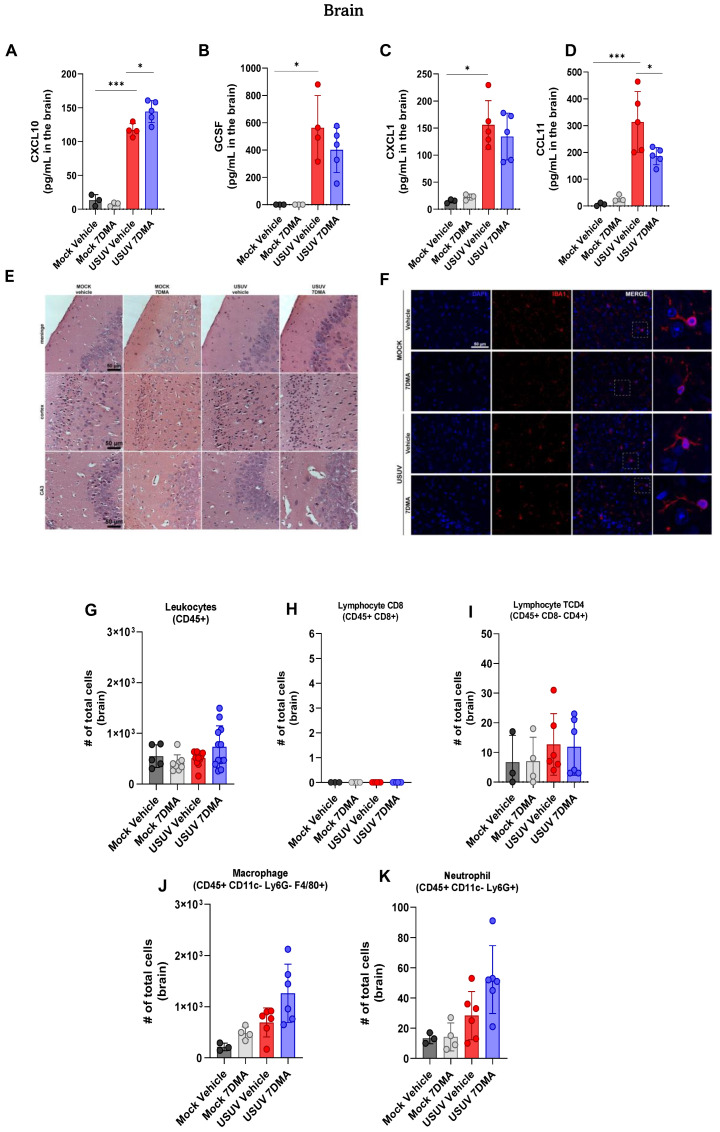
USUV infection of IFNAR^-/-^ deficient mice elicits a limited and ineffective immune response in the brain, and 7DMA treatment is associated with distinct changes in this profile. (**A**–**D**) USUV infection increases levels of cytokines and chemokines in IFNAR^-/-^ brain. (**E**) Histopathology of hippocampal areas and (**F**) anti-Iba1 immunofluorescence staining for microglia showed no morphological alterations in the mouse brain. (**G**–**K**) Flow cytometry analysis showed no difference in leukocyte number in IFNAR^-/-^ brain. Multiplex ELISA of 3 mice per Mock group and 5 mice per infected group. Cytokine and chemokine quantification data were analyzed using two-way ANOVA with Dunnett’s multiple comparison test. Representative images of two independent experiments with 5 individuals per group. Flow cytometry experiment conducted with 8 mice per Mock group and 12 mice per USUV-infected group. Significance was determined by the Kruskal–Wallis test, followed by Dunn’s multiple-comparison test. * *p* < 0.05 and *** *p* < 0.001.

USUV has been demonstrated to be neurotropic in birds and humans [24]. We sought to investigate if USUV invasion of the murine central nervous system (CNS) would also result in the induction of a robust inflammatory response, in association with neurological disease. Analysis of cytokines and chemokines expressed in the brain of IFNAR^-/-^ mice indicated that USUV induced the expression of CXCL10 (Figure 5A), G-CSF (Figure 5B), CXCL1 (Figure 5C) and CCL11 (Figure 5D). 7DMA treatment led to a discrete increase in the expression of CXCL10 and, conversely, to a reduction in the expression of CCL11. Histological analysis of brain sections at day 5 p.i. showed no evidence of significant brain inflammation after USUV infection, observed in the absence of leukocyte recruitment, hemorrhage, vascular alterations, or tissue damage (Figure 5E). Moreover, immunofluorescent staining of IBA-1-expressing cells, indicative of microglia, in mouse brain sections showed that USUV infection did not affect the number or distribution of IBA-1-positive cells (Figure 5F). Observation of IBA-1-positive cells under higher magnification also revealed that microglial morphology was unaffected by USUV infection or by the expression of inflammatory mediators in the brain. 7DMA treatment did not change the histological aspects or IBA-1 staining in the brains of infected or non-infected mice.

Flow cytometry analysis of leukocyte populations recruited to the brain corroborated our histological findings, showing that the total number of CD45^+^ leukocytes (Figure 5G) in the brain was similar between all experimental groups. The number of T CD8^+^ and TCD4^+^ lymphocytes (Figure 5H,I) in mouse brains was at a baseline level. Also, the number of macrophages (Figure 5J) and neutrophils (Figure 5K) recruited to USUV-infected brains was also similar between all experiment groups, though we observed a tendency for an increase in the USUV-infected 7DMA-treated group.

## 4. Discussion

In this study, we investigated the effect of 7DMA treatment over the course of USUV infection in IFNAR^-/-^ mice, focusing on its impact on viral replication and the host immune response. Nucleoside analogues are among the most widely used and effective classes of antiviral drugs, and several compounds have demonstrated broad activity against orthoflaviviruses [25,26,27]. However, despite their potential, no therapies are currently approved for Orthoflavivirus infections. Further studies are needed to define the protective mechanisms of nucleoside analogues and to evaluate their efficacy in relevant animal models. This gap is of particular concern, given the increasing dispersion and re-emergence of arboviruses such as USUV, which highlights the urgency of developing effective treatments for these neglected pathogens.

To evaluate the therapeutic effects of 7DMA during peak infection, tissue samples were collected at day 5 post-infection which corresponds to the peak of infection in this model. This timepoint was specifically selected to enable a paired analysis of the effects of 7DMA treatment in comparison to the infected, untreated group, which uniformly succumbs to infection by day 6. As previously shown by our group, an inoculum as low as 10^1^ PFU is sufficient to induce 100% mortality in wild-type mice [24]. In the present study, we demonstrate that 7DMA remains effective even when immunosuppressed mice are infected with a higher viral dose (10^4^ PFU), highlighting the potency of the compound under highly stringent conditions.

Because treatment was discontinued at day 6 post-infection, we were unable to evaluate long-term outcomes or assess post-treatment viral rebound, which limits our ability to fully characterize the durability of 7DMA’s protective effects. This question is beyond the scope of the present study; however, it remains an important avenue for future investigation and underscores the need for complementary studies in less acute or immunocompetent models to better assess the long-term efficacy, resistance potential, and therapeutic applicability of 7DMA.

Following subcutaneous infection of IFNAR^-/-^ mice with USUV, we observed that the virus rapidly reached the brain at early time points. In contrast, Samuel et al. demonstrated that WNV-infected IFNAR^-/-^ mice developed markedly elevated splenic viral loads by day 2 post-infection, reaching levels up to 3 logs higher than those in serum and brain, accompanied by disruption of splenic architecture and reduced cellularity [28]. These findings highlight distinct dissemination patterns among related orthoflaviviruses and underscore the role of type I interferons in shaping viral tropism in peripheral tissues, while also emphasizing the strong neurotropic capacity of our USUV strain [29].

Despite the increased viral load detected in the brain, no histopathological evidence of neuronal death, leukocyte infiltration, or microglial activation was observed, based on microscopic analysis of hippocampal sections stained solely with H&E. This is consistent with the model developed by Kuchinsky et al., in which IFNAR^-/-^ mice presented no morphological evidence of inflammation or cell death in cortical sections, while the most pronounced alterations were instead observed in the spleen [23]. However, neuronal cell damage cannot be ruled out in these models, given that neither study employed specific methods to detect it.

The lack of observable brain damage in H&E-stained sections from infected IFNAR^-/-^ mice aligns with evidence that type I IFN signaling impacts the local inflammatory milieu in the brain, leading to the loss of specific cellular cues required for microglial activation [30] and thereby rendering mice more susceptible to infection. Accordingly, while intracranial infection of IFNAR^-/-^ mice with St. Louis encephalitis virus (SLEV) induces much higher expression of proinflammatory cytokines such as IL-1β, CCL5, and CXCL1 compared to infected wild-type (WT) mice, this occurs without microglial activation or significant recruitment of leukocytes to the brain [31]. Moreover, USUV infection in suckling mice, which lack a fully developed adaptive immune system but retain type I interferon responses, results in pronounced CNS inflammation.

In this USUV infection model, cytokines CXCL10, CCL11, CXCL1, and G-CSF peaked in the brain at later time points. These cytokines have been linked to neuroinflammation in USUV-infected adult immunocompetent mice following intracranial inoculation, where they contribute to Th1 polarization and monocyte recruitment to the brain [32]. However, their upregulation in those WT mice takes place within a broader neuroinflammatory response, marked by the induction of many additional cytokines and widespread brain inflammation. In contrast, several key cytokines were not induced in the brains of USUV-infected IFNAR^-/-^ mice, notably IFN-γ. In USUV infection, IFN-γ is shown to play an important neuroregulatory role by driving CCR2-dependent monocyte recruitment and microglial activation after intracranial infection in adult immunocompetent mice [31,33]. Although our study did not directly investigate how type I interferon signaling shapes neuroinflammation, the absence of leukocyte infiltration, microglial activation, and IFN-γ expression in the brain supports previous findings by underscoring the critical role of IFN-γ in coordinating immune cell recruitment and driving inflammatory responses during USUV infection.

Guerrero et al. evaluated the efficacy of several nucleoside analogues, including 2′-C-methyladenosine (2′CMA) and Ribavirin ex vivo and identified 7DMA as one of the most effective options against USUV infection [34]. Their study primarily focused on favipiravir and showed that its antiviral activity was most pronounced within the first 6 h post-infection in Vero E6 cells. Although 7DMA showed only a trend toward reducing viral load in Vero CCL-81 cells between 4 and 6 h post-infection (*p* = 0.08 at 4 h; *p* = 0.09 at 6 h), this may reflect the higher replication capacity typically observed in CCL-81 cells when compared to Vero E6 cells, as well as differences in methodology, with their study using qPCR to detect viral RNA and ours using plaque assays to measure infectious viral particles. Importantly, in neuroblastoma cells, 7DMA effectively suppressed USUV replication within the first 6 h, matching the antiviral activity of favipiravir. This is consistent with the expected mechanism of this class of nucleoside analogues, which inhibit viral RNA synthesis during the early stages of infection. Notably, because 7DMA was administered for a short period, we did not evaluate the emergence of resistance-associated variants, as our conditions did not entail prolonged exposure or sustained drug pressure required for their selection [16,35]. This analysis was beyond the scope of the present study and warrants future investigation.

The AG129 mouse model, which lacks both type I and type II interferon receptors, was used to assess the antiviral activity of favipiravir [34]. In this model, favipiravir delayed mortality and reduced viral replication; however, the mechanisms underlying this effect were not further investigated. Consistent with their findings, we show that treatment with 7DMA reduced viral loads not only in the brain but also in the serum and peripheral tissues of IFNAR^-/-^ mice. While the antiviral effect of 7DMA was already evident by day 3, when the virus was detected in the serum and brain, it remains unclear whether this reduction reflects a direct effect within the CNS and other tissues or an indirect consequence of reduced systemic viremia.

Importantly, treatment reduced CCL11 and increased CXCL10 levels in the brains of infected mice. Interestingly, CXCL10 was also elevated in peripheral tissues, whereas CCL11 expression remained restricted to the brain. The cellular source of CCL11 in the brain remains to be determined, as the mechanism by which 7DMA can lead to a reduction in the brain levels of this chemokine while increasing others. While we did not directly assess whether CCL11 levels would rebound at later time points after treatment, uninfected, untreated animals displayed a marked increase in this cytokine before rapidly succumbing to infection. CCL11 is widely recognized as a biomarker of neuroinflammatory disorders, including multiple sclerosis, Parkinson’s disease, Alzheimer’s disease, and COVID-19–associated neurological syndromes [36], and warrants further investigation into its involvement in USUV-associated neuropathogenesis.

Although brain inflammation was limited in our model, cytokines, including G-CSF, CXCL10, CXCL1, IL-6, and IL-1β, were already increased in the periphery by day 3 post-infection, despite a low to undetectable viral load. This systemic response was followed by an increase in additional inflammatory cytokines such as LIF, GM-CSF, and IL-15 at the peak of infection. Interestingly, treatment with 7DMA further reshaped this profile in the spleen: G-CSF levels were reduced, whereas CXCL10, GM-CSF, IL-15, and IL-1β were increased relative to untreated animals. Notably, IL-15 and GM-CSF have been shown to promote cytoskeletal rearrangements in neutrophils, thereby enhancing tissue migration [37]. Consistent with this finding, infection increased splenic neutrophil numbers in both infected and treated mice. Although treatment did not alter overall neutrophil abundance, it shifted their phenotype by modifying the proportion of CD44^+^ neutrophils. While we did not directly assess neutrophil activation, this observation aligns with findings from Gerritje et al., who reported that CD44 deficiency resulted in elevated cytokine levels such as IL-6, IL-1β, and CXCL1 [38], all of which were also increased in our model.

Finally, we observed a reduction in splenic lymphocyte populations in both mock- and infected groups treated with 7DMA. In vivo cytotoxicity is a known effect of nucleoside analogues, supported by a previous report linking 7DMA to mitochondrial toxicity [39]. Such toxicity has been associated with elevated IL-6 levels and caspase activation, which can impair CD8^+^ T-cell survival through mitochondrial dysfunction and ROS production. Although we did not directly investigate this mechanism or 7DMA acute toxicity in our in vivo infection model, we hypothesize that the reduction in splenic lymphocyte numbers may be related to 7DMA toxic effects, underscoring the need for further evaluation of 7DMA safety in vivo.

In summary, our study demonstrates that treatment with the nucleoside analogue 7DMA delays disease progression, reduces viral loads, and is associated with alterations in the inflammatory profile across tissues, including decreased levels of CCL11 in the brain and reduced neutrophil activation in the periphery. These findings underscore the importance of further investigating the role of innate immune pathways in USUV pathogenesis and support 7DMA as a potential antiviral candidate. While 7DMA may also influence host immune responses, additional studies are needed to clarify whether these effects are direct or secondary to reduced viral replication. This provides a rationale for the development of novel therapeutic strategies for Orthoflavivirus infections.

## Figures and Tables

**Table 1:** Antibodies for immunophenotyping tissue-isolated cells.

Specificity	Clone	Fluorochrome	Dilution	ID	Supplier
CD16/32	93	-	1:100	101301	BioLegend
CD8a	53-6.7	PE	1:400	553032	BD Bioscience
CD4	RM4-5	FITC	1:200	553046	BD Bioscience
CD11c	N418	BUV805	1:100	749038	BD Bioscience
CD11b	M1/70	ALEXA 488	1:800	557672	BD Bioscience
CD45	30-F11	BUV563	1:400	612924	BD Bioscience
CD25	3C7	APC	1:200	558643	BD Bioscience
F4/80	T45-2342	APC	1:200	566787	BD Bioscience
LY-6G	1A8	PE-CY7	1:200	560601	BD Bioscience
CD44	IM7	BV711	1:200	563971	BD Bioscience

## Data Availability

All data used in this study has been included in the manuscript. Raw data files can be provided upon request.

## Supplementary Materials

The following supporting information can be downloaded at: https://www.mdpi.com/article/10.3390/v17121639/s1, Figure S1: Multiplex quantification of cytokine and chemokine levels in IFNAR^-/-^ mouse brain, liver and spleen during the course of USUV infection and 7DMA treatment; Figure S2: Quantification of cytokines and chemokines in IFNAR^-/-^ mice on day 3 post USUV infection and 7DMA treatment; Table S1: Descriptive data of multiplex of spleen; Table S2: Descriptive data of multiplex of liver; Table S3: Descriptive data of multiplex of brain; Figure S3: Gating strategy for lymphocytes and myeloid cells in spleen; Figure S4: Gating strategy for lymphocytes and myeloid cells in blood; Figure S5: Gating strategy for lymphocytes and myeloid cells in brain.
