# Supplementary material for: Antiviral and Immunomodulatory Effects of 7-Deaza-2-methyladenosine (7DMA) in a Susceptible Mouse Model of Usutu Virus Infection
Source: Viruses. 2025 Dec 18;17(12):1639. doi: 10.3390/v17121639 (PMC12737359; doi:10.3390/v17121639)

SUPPLEMENTARY FIGURE 1

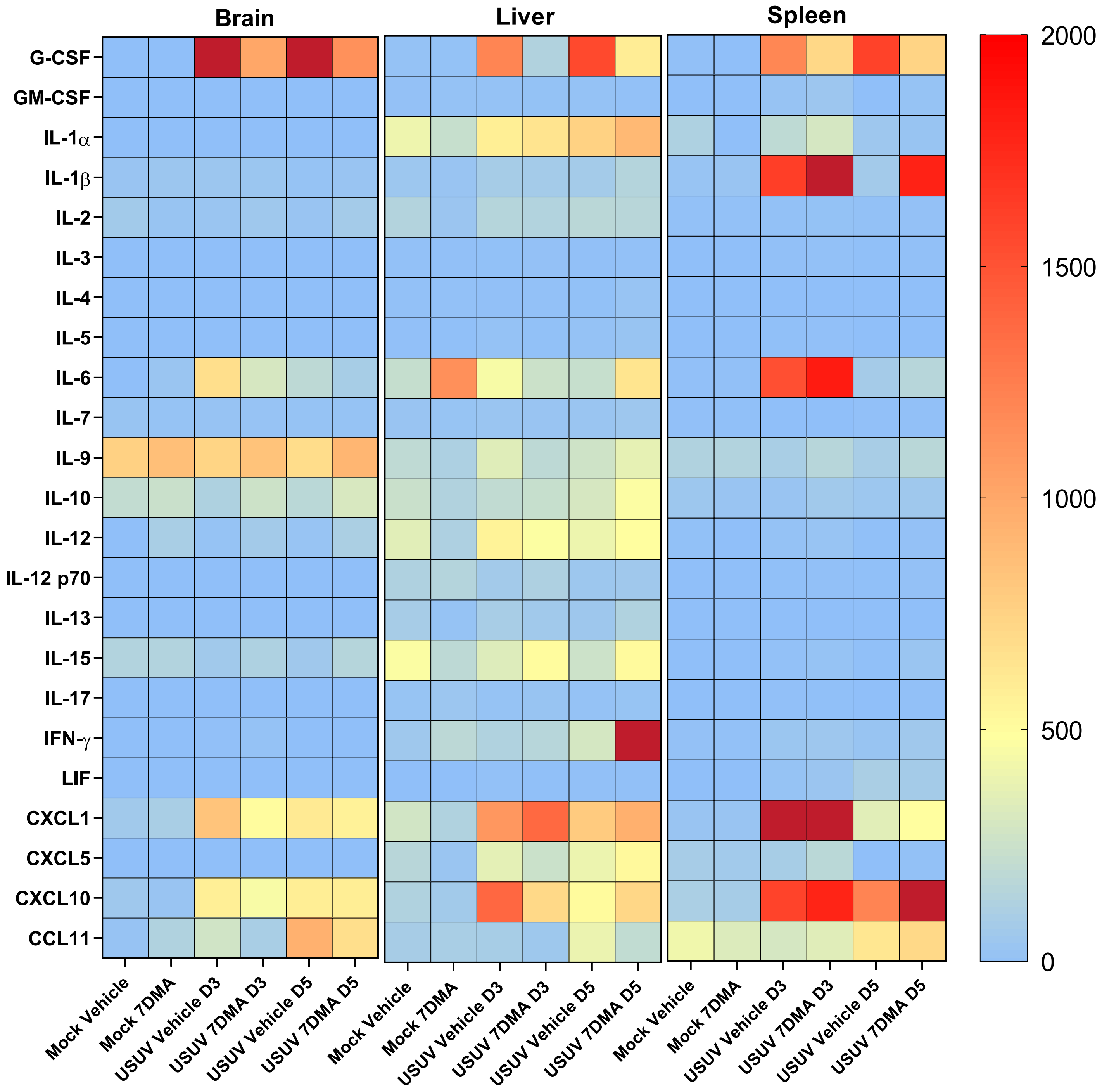

SUPPLEMENTARY FIGURE 2

Spleen
Day 3 post-infection

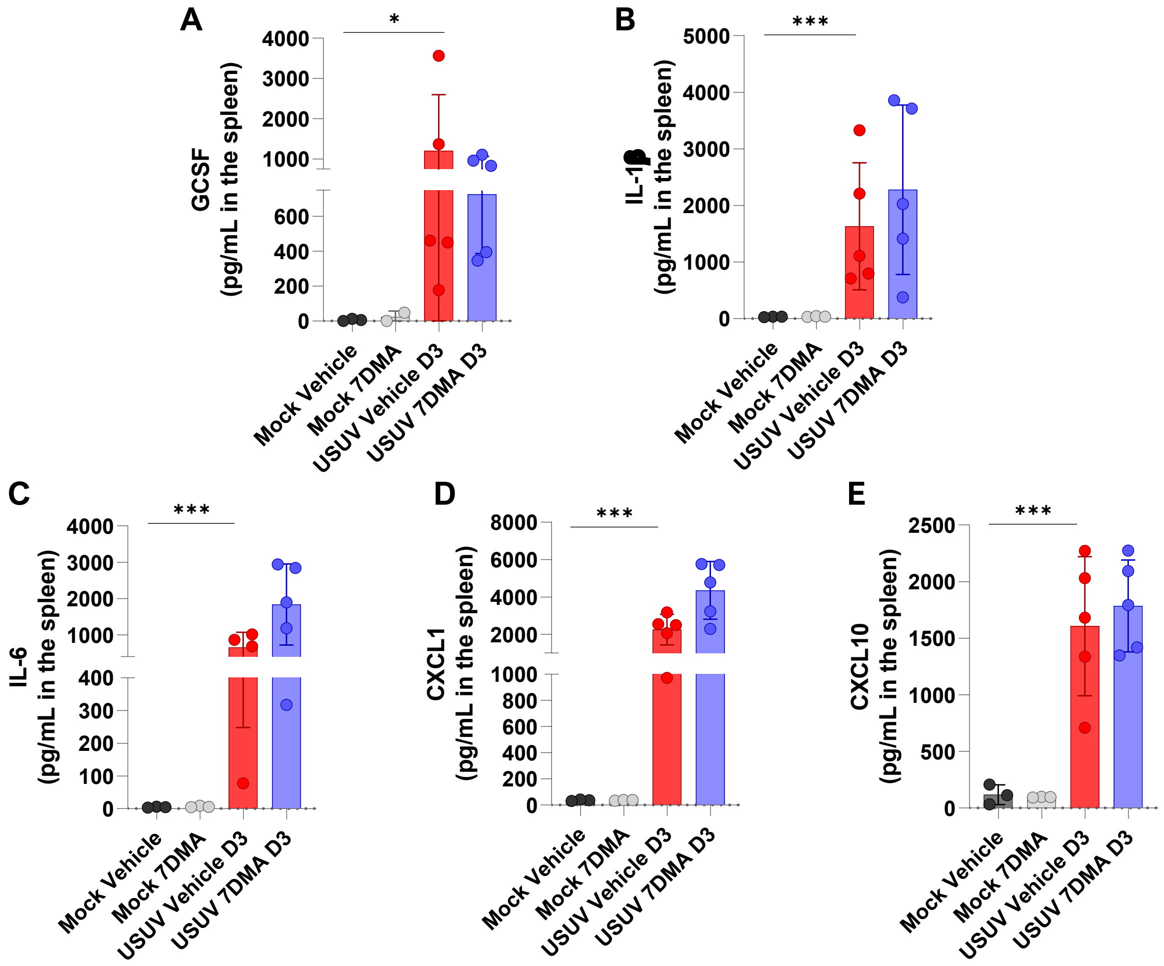

Liver
Day 3 post-infection

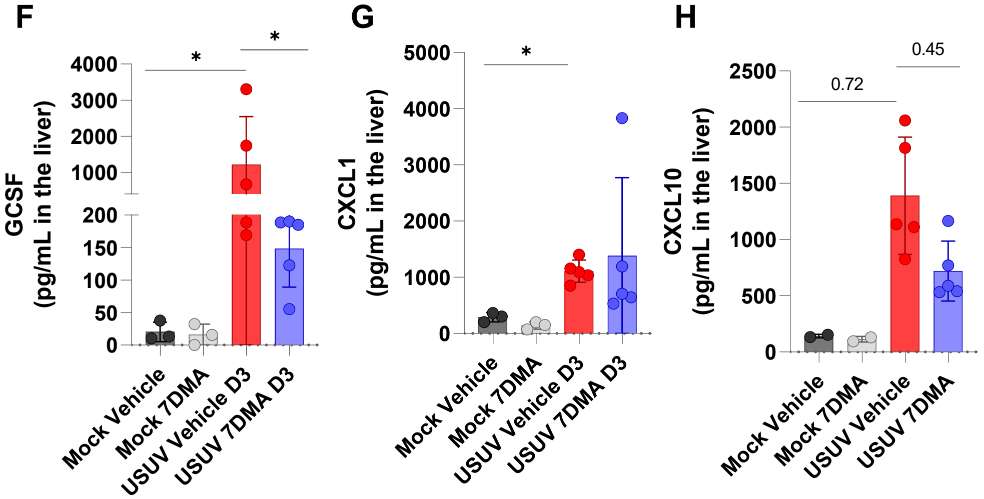

Brain
Day 3 post-infection

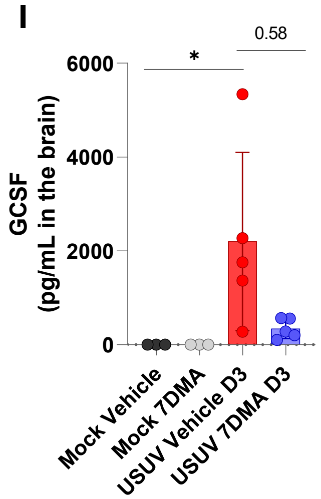

SUPPLEMENTARY TABLE 1

SPLEEN

Cytokine	Group	n	Min.	Max.	Mean	SD	p value (Treatment)	p value (Infection)	p value (Inf. x Treat.)
**G-CSF**	Mock	3	0.0	12.7	6,3	6,4			
	Mock 7DMA	2	0.0	48.0	24,0	33.94	0.99		
	USUV Vehicle (D3)	5	178,0	3563,3	1204.0	1393.3		**0.004**	
	USUV 7DMA (D3)	5	346,3	1101,9	726.1	338.7			0.47
	USUV Vehicle (D5)	4	1180.0	99.67	1180.0	99.67		**0.0001**	
	USUV 7DMA (D5)	5	526.1	1393.1	748,4	382,6			**0.002**
**GM-CSF**	Mock	3	0,0	0,0	0,0	0,0			
	Mock 7DMA	3	0,0	0,0	0,0	0,0	>0.99		
	USUV Vehicle (D3)	5	0,0	46,3	30.5	19.5		>0.99	
	USUV 7DMA (D3)	5	6,3	102,5	54.6	34.5			>0.99
	USUV Vehicle (D5)	5	0,0	0,0	0,0	0,0			
	USUV 7DMA (D5)	5	6.3	44.1	23,9	14,2		>0.99	**0.003**
**IL-1α**	Mock	3	0.0	365.4	133,4	201,6			
	Mock 7DMA	3	0.0	0.0	0,0	0,0	0.94		
	USUV Vehicle (D3)	5	0,0	397,9	206.7	161.3		0.99	
	USUV 7DMA (D3)	5	131,8	452,8	308.2	122.0			0.99
	USUV Vehicle (D5)	5	0.0	145.1	61,0	64,0		0.98	
	USUV 7DMA (D5)	5	0.0	126.3	36,0	55,6			0.99
**IL-1β**	Mock	3	25.2	37.4	32,3	6,3			
	Mock 7DMA	3	32.4	44.5	36,8	6,7	>0.99		
	USUV Vehicle (D3)	5	708,4	3329	1630.6	1122.3		**<0.0001**	
	USUV 7DMA (D3)	5	337,9	3858,2	2277.7	1497.4			0.14
	USUV Vehicle (D5)	5	68.5	100.5	83,6	14,3		0.97	
	USUV 7DMA (D5)	5	221.1	6846.7	1794,8	2848,0			**<0.0001**
**IL-2**	Mock	2	8.2	14.1	11,0	3,0			
	Mock 7DMA	3	6.1	14.0	9,6	4,0	>0.99		
	USUV Vehicle (D3)	5	4,1	20,1	13.4	5.8		>0.99	
	USUV 7DMA (D3)	5	11,1	23,6	17.8	5.0			>0.99
	USUV Vehicle (D5)	5	8.6	16.3	12,9	3,3		>0.99	
	USUV 7DMA (D5)	5	9.7	24.6	15,3	5,6			>0.99
**IL-3**	Mock	3	0.0	0.0	0,0	0,0			
	Mock 7DMA	3	0.0	0.0	0,0	0,0	>0.99		
	USUV Vehicle (D3)	5	0,0	13,4	7.6	5.6		>0.99	
	USUV 7DMA (D3)	5	8,0	13,6	10.1	2.5			>0.99
	USUV Vehicle (D5)	3	3.7	4.1	3,9	0,3		>0.99	
	USUV 7DMA (D5)	5	3.6	13.40	7,6	4,8			>0.99
**IL-4**	Mock	3	0.0	0.0	0.0	0.0			
	Mock 7DMA	3	0.0	0.0	0.0	0.0	>0.99		
	USUV Vehicle (D3)	5	0.0	0.0	0.0	0.0		>0.99	
	USUV 7DMA (D3)	5	0.0	0.0	0.0	0.0			>0.99
	USUV Vehicle (D5)	5	0.0	0.0	0.0	0.0		>0.99	
	USUV 7DMA (D5)	5	0.0	0.0	0.0	0.0			>0.99
**IL-5**	Mock	3	0.0	0.0	0.0	0.0			
	Mock 7DMA	3	0.0	0.0	0.0	0.0	>0.99		
	USUV Vehicle (D3)	5	0.0	0.0	0.0	0.0		>0.99	
	USUV 7DMA (D3)	5	0.0	0.0	0.0	0.0			>0.99
	USUV Vehicle (D5)	5	0.0	0.0	0.0	0.0		>0.99	
	USUV 7DMA (D5)	5	0.0	0.0	0.0	0.0			>0.99
**IL-6**	Mock	2	3.3	6.4	4,7	1,6			
	Mock 7DMA	3	4.8	8.9	6,4	2,2	>0.99		
	USUV Vehicle (D3)	5	77,4	5020,3	1531,1	1982,7		**<0.0001**	
	USUV 7DMA (D3)	5	317,2	2942	1835,5	1115,2			0.80

	USUV Vehicle (D5)	5	65.6	102.1	92,2	29,7		**0.03**	
	USUV 7DMA (D5)	5	87.6	493.1	172,5	179,4			0.97
**IL-7**	Mock	1	0,0	0,0	5,5	1,7			
	Mock 7DMA	3	0,0	0,0	4,3	0,4	>0.99		
	USUV Vehicle (D3)	5	0,0	0,0	0,0	0,0		>0.99	
	USUV 7DMA (D3)	5	0,0	0,0	0,0	0,0			>0.99
	USUV Vehicle (D5)	5	0,0	0,0	4,7	1,1		>0.99	
	USUV 7DMA (D5)	5	0,0	0,0	6,9	1,1			>0.99
**IL-9**	Mock	3	125.4	174.1	143,1	26,9			
	Mock 7DMA	3	120.9	181.6	149,0	30,6	>0.99		
	USUV Vehicle (D3)	5	37,7	160,7	111,2	50,9		0.99	
	USUV 7DMA (D3)	5	129,2	286,4	171,9	65,1			0.99
	USUV Vehicle (D5)	5	45.8	146.7	106,5	42,3		0.99	
	USUV 7DMA (D5)	5	129.8	257.5	184,7	48,3			0.98
**IL-10**	Mock	3	32.6	96.9	56,4	35,3			
	Mock 7DMA	3	24.5	45.2	37,6	11,4	0.99		
	USUV Vehicle (D3)	5	5,7	54,4	36,2	18,5		>0.99	
	USUV 7DMA (D3)	5	38,6	127,8	74,5	34,6			>0.99
	USUV Vehicle (D5)	5	35.4	82.6	55,2	18,1		>0.99	
	USUV 7DMA (D5)	5	51.0	80.4	66,4	14,6			>0.99
**IL-12**	Mock	2	0.0	0.0	8,6	15,0			
	Mock 7DMA	3	0.0	0.0	0,0	0,0	>0.99		
	USUV Vehicle (D3)	5	0.0	19,8	18,5	19,0		>0.99	
	USUV 7DMA (D3)	5	0.0	52,7	32,6	20,6			>0.99
	USUV Vehicle (D5)	5	0.0	7.7	1,5	3,4		>0.99	
	USUV 7DMA (D5)	5	0.0	37.8	15,2	15,9			0.30
**IL-12 p70**	Mock	2	0.0	0.0	0.0	0.0			
	Mock 7DMA	3	0.0	0.0	0.0	0.0	>0.99		
	USUV Vehicle (D3)	5	0,0	9,8	3,3	5,7		>0.99	
	USUV 7DMA (D3)	5	0,0	12,7	6,1	5,5			>0.99
	USUV Vehicle (D5)	4	0.0	16.0	5.8	7.6		>0.99	
	USUV 7DMA (D5)	5	5.8	25.3	14,1	8,2			0.39
**IL-13**	Mock	3	0.0	0.0	0.0	0.0			
	Mock 7DMA	3	0.0	0.0	0.0	0.0	>0.99		
	USUV Vehicle (D3)	5	0.0	0.0	0.0	0.0		>0.99	
	USUV 7DMA (D3)	5	0.0	0.0	0.0	0.0			>0.99
	USUV Vehicle (D5)	5	0.0	0.0	0.0	0.0		>0.99	
	USUV 7DMA (D5)	5	0.0	0.0	0.0	0.0			>0.99
**IL-15**	Mock	3	0.0	0.0	0.0	0.0			
	Mock 7DMA	3	0.0	0.0	0.0	0.0	>0.99		
	USUV Vehicle (D3)	5	0,0	30,4	10,9	12,7		0.99	
	USUV 7DMA (D3)	5	3,9	32,7	23,7	11,8			0.99
	USUV Vehicle (D5)	5	0.0	3.9	0,8	1,7		>0.99	
	USUV 7DMA (D5)	5	19.3	53.7	41,5	13,5			**0.01**
**IL-17**	Mock	3	0.0	0.0	0.0	0.0			
	Mock 7DMA	3	0.0	0.0	0.0	0.0	>0.99		
	USUV Vehicle (D3)	5	3,9	5,4	4,7	1,1		>0.99	
	USUV 7DMA (D3)	5	3,3	4,7	4,1	0,7			>0.99
	USUV Vehicle (D5)	5	0.0	0.0	0.0	0.0		>0.99	
	USUV 7DMA (D5)	5	0.0	0.0	0.0	0.0			>0.99
**IFN-γ**	Mock	3	6.3	23.9	15,2	8,8			
	Mock 7DMA	3	0.0	22.4	10,9	11,2	>0.99		
	USUV Vehicle (D3)	5	37,3	69,8	47,9	13,3		0.99	
	USUV 7DMA (D3)	5	18,3	126,4	59,3	40,9			>0.99
	USUV Vehicle (D5)	5	23.2	48.8	35,8	10,8		0.20	
	USUV 7DMA (D5)	5	18.4	207.3	68,9	78,7			0.99
**LIF**	Mock	3	0.0	0.0	0.0	0.0			

	Mock 7DMA	3	0.0	0.0	0.0	0.0	>0.99		
	USUV Vehicle (D3)	5	20,5	29,0	24,6	4,0		>0.99	
	USUV 7DMA (D3)	5	8,2	75,4	45,8	25,1			>0.99
	USUV Vehicle (D5)	5	82.30	130.3	114,4	19,1		**0.01**	
	USUV 7DMA (D5)	5	65.70	108.6	90.02	17,9			0.58
**CXCL1**	Mock	3	31.4	42.9	37,4	5,8			
	Mock 7DMA	3	33.3	39.4	36,9	3,2	>0.99		
	USUV Vehicle (D3)	5	971,2	3180,6	2253,0	819,3		**<0.0001**	
	USUV 7DMA (D3)	5	2291,7	5765,2	4352,1	1542,4			**<0.0001**
	USUV Vehicle (D5)	5	236.2	439.0	364,8	86,1		0.57	
	USUV 7DMA (D5)	5	263.2	991.7	500,6	289,5			0.91
**CXCL5**	Mock	3	48.60	158.3	100,6	55,1			
	Mock 7DMA	3	63.60	95.00	78,6	15,8	0.99		
	USUV Vehicle (D3)	5	0,0	184,9	101,7	71,3		>0.99	
	USUV 7DMA (D3)	5	117,7	231,3	187,7	42,8			0.99
	USUV Vehicle (D5)	5	0.0	0.0	0.0	0.0		**0.02**	
	USUV 7DMA (D5)	4	0.0	0.0	0.0	0.0			>0.99
**CXCL10**	Mock	3	32.0	208.0	117,6	88,1			
	Mock 7DMA	3	92.3	99.8	96,3	3,8	0.99		
	USUV Vehicle (D3)	5	708,5	2271,3	1605,6	613,5		**<0.0001**	
	USUV 7DMA (D3)	5	1347,2	2274,6	1785,2	406,5			0.61
	USUV Vehicle (D5)	5	956.9	1364.4	1220,6	160,4		**0.001**	
	USUV 7DMA (D5)	5	1419.8	5023.3	2316,2	1521,8			**<0.001**
**CCL11**	Mock	3	289.3	509.7	431,1	123,0			
	Mock 7DMA	3	269.2	430.4	339,1	82,7	>0.99		
	USUV Vehicle (D3)	5	88,1	492	307,1	177,4		0.99	
	USUV 7DMA (D3)	5	273,8	471,2	356,8	72,2			0.74
	USUV Vehicle (D5)	5	545.5	733.9	637,6	70,4		0.21	
	USUV 7DMA (D5)	5	588.1	837.5	718,5	123,3			>0.99

SUPPLEMENTARY TABLE 2

LIVER

Cytokine	Group	n	Min.	Max.	Mean	SD	p value (Treatment)	p value (Infection)	p value (Inf. x Treat.)
**G-CSF**	Mock	3	10.2	37.5	20.23	15.01			
	Mock 7DMA	3	0.0	32.4	16.03	16.20	>0.99		
	USUV Vehicle (D3)	5	169,3	3300,7	1212.7	1329.4		**0.01**	
	USUV 7DMA (D3)	5	55,4	190,2	148.4	59.2			**0.01**
	USUV Vehicle (D5)	4	636.2	3359.0	1569.06	1073.61		**0.01**	
	USUV 7DMA (D5)	5	97.6	1232.1	600.44	435.96			**0.01**
**GM-CSF**	Mock	3	6.3	25.8	15.16	9.89			
	Mock 7DMA	2	22.3	37.2	29.75	10.53	>0.99		
	USUV Vehicle (D3)	5	8,6	27,4	20.5	7.1		>0.99	
	USUV 7DMA (D3)	5	10,4	27,4	17.2	7.0			>0.99
	USUV Vehicle (D5)	5	0.0	31.9	19.04	12.56		>0.99	
	USUV 7DMA (D5)	5	0.0	16.0	9.22	6.29			>0.99
**IL-1α**	Mock	3	196.9	720.0	420.53	269.66			
	Mock 7DMA	3	79.7	326.7	242.50	141.01	>0.99		
	USUV Vehicle (D3)	5	412,0	988,8	593.9	254.7		0.97	
	USUV 7DMA (D3)	5	117,5	1288,5	653.3	421.9			>0.99
	USUV Vehicle (D5)	5	637.6	957.8	763.80	131.54		0.99	
	USUV 7DMA (D5)	4	647.9	1219.7	911.32	264.23			>0.99
**IL-1β**	Mock	3	50.5	60.2	54.80	4.94			
	Mock 7DMA	2	52.7	58.2	55.45	3.88	>0.99		
	USUV Vehicle (D3)	5	73	112,7	96.2	16.7		0.99	
	USUV 7DMA (D3)	5	75,1	105,3	89.0	10.8			>0.99
	USUV Vehicle (D5)	5	66.0	116.6	89.52	19.50		>0.99	
	USUV 7DMA (D5)	5	105.3	189.2	159.24	31.83			>0.99
**IL-2**	Mock	3	138.5	178.3	155.36	20.58			
	Mock 7DMA	2	12.0	80.5	46.25	48.43	>0.99		
	USUV Vehicle (D3)	5	131,9	200,3	166.2	33.2		>0.99	
	USUV 7DMA (D3)	5	108,8	177,4	148.9	47.4			>0.99
	USUV Vehicle (D5)	5	134.3	236.7	185.48	44.24		>0.99	
	USUV 7DMA (D5)	4	72.4	299.5	176.80	96.89			>0.99
**IL-3**	Mock	3	6.0	16.90	10.20	5.86			
	Mock 7DMA	3	0.0	16.20	7.06	8.29	>0.99		
	USUV Vehicle (D3)	5	3,7	11,4	8.7	4.9		>0.99	
	USUV 7DMA (D3)	5	6,7	26,8	14.8	8.8			>0.99
	USUV Vehicle (D5)	5	0.0	11.40	6.10	4.18		>0.99	
	USUV 7DMA (D5)	3	4.90	17.30	10.13	6.42			>0.99
**IL-4**	Mock	3	7.8	11.7	10.26	2.14			
	Mock 7DMA	2	9.4	9.5	9.45	0.07	>0.99		
	USUV Vehicle (D3)	5	3,8	9,2	6.5	2.1		>0.99	
	USUV 7DMA (D3)	5	7,2	15,6	9.9	3.3			>0.99
	USUV Vehicle (D5)	4	6.6	11.8	8.12	2.46		>0.99	
	USUV 7DMA (D5)	4	3.9	14.9	8.9	4.67			>0.99
**IL-5**	Mock	3	0.0	9.0	4,5	4,5			
	Mock 7DMA	3	0.0	5.9	3,1	3,0	>0.99		
	USUV Vehicle (D3)	5	9,5	37,4	20.4	11.6		>0.99	
	USUV 7DMA (D3)	5	0,0	20,6	8,0	7.6			>0.99
	USUV Vehicle (D5)	5	0.0	47.4	22,0	16,9		>0.99	
	USUV 7DMA (D5)	5	11.7	66.5	35,5	24,7			>0.99
**IL-6**	Mock	3	145.5	325.7	236,8	90,1			
	Mock 7DMA	2	41.5	169.6	105,6	90,6	>0.99		
	USUV Vehicle (D3)	5	311,8	754,4	463.3	184.2		0.92	
	USUV 7DMA (D3)	5	206,7	329,1	264.7	51.9			0.91

	USUV Vehicle (D5)	5	176.0	370.8	242,2	76,7		>0.99	
	USUV 7DMA (D5)	4	181.5	1798.9	224.5	108.9			0.95
**IL-7**	Mock	3	16.8	51.4	36,3	17,7			
	Mock 7DMA	3	28.0	76.0	46,2	25,9	>0.99		
	USUV Vehicle (D3)	5	26,4	62,1	44.4	16.6		>0.99	
	USUV 7DMA (D3)	5	20,6	71,3	38.6	19.1			>0.99
	USUV Vehicle (D5)	5	36.1	51.4	41,4	6,8		>0.99	
	USUV 7DMA (D5)	5	30.0	102.0	58,8	29,0			>0.99
**IL-9**	Mock	3	162.6	240.0	207,6	40,2			
	Mock 7DMA	2	170.3	208.3	189,3	26,9	>0.99		
	USUV Vehicle (D3)	5	274,4	449,9	355.8	68.2		0.98	
	USUV 7DMA (D3)	5	148,7	274,4	200.6	61.3			0.96
	USUV Vehicle (D5)	5	188.9	464.6	276,7	111,2		>0.99	
	USUV 7DMA (D5)	5	259.0	553.8	384,0	143,9			>0.99
**IL-10**	Mock	3	203.2	326.3	256,1	63,3			
	Mock 7DMA	2	183.9	249.8	216,9	46,6	>0.99		
	USUV Vehicle (D3)	5	146,2	299,1	216.6	60.9		0.99	
	USUV 7DMA (D3)	5	182,1	294,6	243.1	47.3			0.99
	USUV Vehicle (D5)	5	232.9	380.6	311,6	63,9		>0.99	
	USUV 7DMA (D5)	5	326.5	710.7	482,8	145,9			>0.99
**IL-12**	Mock	3	306.7	435.9	362,0	66,6			
	Mock 7DMA	3	0.0	330.0	131,6	174,8	>0.99		
	USUV Vehicle (D3)	5	183,3	1335,4	570.2	451.0		0.94	
	USUV 7DMA (D3)	5	277,2	667,8	485.7	169.1			0.97
	USUV Vehicle (D5)	5	252.7	594.7	418,3	137,3		>0.99	
	USUV 7DMA (D5)	5	32.7	835.6	503,7	315,5			>0.99
**IL-12 p70**	Mock	3	69.0	200.4	137,8	65,9			
	Mock 7DMA	2	114.4	367.1	240,8	178,7	>0.99		
	USUV Vehicle (D3)	5	29,6	119,2	82.1	35.5		0.99	
	USUV 7DMA (D3)	5	83,7	243,6	131.0	75.0			0.99
	USUV Vehicle (D5)	5	0.0	90.1	57,2	36,6		>0.99	
	USUV 7DMA (D5)	5	20.0	125.8	69,5	38,0			>0.99
**IL-13**	Mock	3	64.1	157.4	100,6	49,9			
	Mock 7DMA	2	25.8	53.4	39,6	19,5	>0.99		
	USUV Vehicle (D3)	5	58,5	240,9	103.9	79.3		0.99	
	USUV 7DMA (D3)	5	47,2	95,1	75.0	20.4			0.99
	USUV Vehicle (D5)	5	44.1	93.0	62,2	19,7		>0.99	
	USUV 7DMA (D5)	5	110.5	167.4	143,9	26,2			>0.99
**IL-15**	Mock	3	412.9	559.4	481,6	73,7			
	Mock 7DMA	2	0.0	399.9	200,0	282,8	>0.99		
	USUV Vehicle (D3)	5	286,8	410,5	343.9	62.8		0.99	
	USUV 7DMA (D3)	5	282,9	757,6	515.6	201.4			0.9
	USUV Vehicle (D5)	5	181.3	331.7	268,1	58,2		>0.99	
	USUV 7DMA (D5)	5	243.6	842.7	523,9	250,6			>0.99
**IL-17**	Mock	3	17.1	38.7	31,4	12,4			
	Mock 7DMA	3	24.7	115.0	71,6	45,2	>0.99		
	USUV Vehicle (D3)	5	20,1	37,7	26.4	8.0		>0.99	
	USUV 7DMA (D3)	5	17,4	52,9	34.8	14.8			>0.99
	USUV Vehicle (D5)	5	5.9	34.0	20,9	10,3		>0.99	
	USUV 7DMA (D5)	5	14.2	49.9	31,4	13,1			>0.99
**IFN-γ**	Mock	3	37.6	92.0	65,7	27,2			
	Mock 7DMA	3	19.6	492.0	194,8	258,8	>0.99		
	USUV Vehicle (D3)	5	10,4	84,1	38,3	31,8		>0.99	
	USUV 7DMA (D3)	5	44,0	186,2	172.9	130.2			>0.99
	USUV Vehicle (D5)	4	3.4	109.8	57,7	43,5		>0.99	
	USUV 7DMA (D5)	5	100.8	7059.5	2014,1	2989,1			**<0.0001**
**LIF**	Mock	3	0.0	0.0	0,0	0,0			

	Mock 7DMA	2	0.0	0.0	0,0	0,0	>0.99		
	USUV Vehicle (D3)	5	7,6	133,0	68,6	56,4		>0.99	
	USUV 7DMA (D3)	5	0,0	158,0	60,1	64,7			>0.99
	USUV Vehicle (D5)	5	0.0	11.70	7,1	4,3		>0.99	
	USUV 7DMA (D5)	4	6.0	8.60	7,5	1,2			>0.99
**CXCL1**	Mock	3	200.1	365.5	289,1	83,4			
	Mock 7DMA	3	73.8	202.7	142,6	64,9	>0.99		
	USUV Vehicle (D3)	5	852,3	1402,3	1108.0	199.6		**0.02**	
	USUV 7DMA (D3)	5	532,1	3830,7	1381.2	1392.3			**0.75**
	USUV Vehicle (D5)	5	592.6	1042.7	803,0	197,4		**0.02**	
	USUV 7DMA (D5)	5	620.0	1538.5	963,5	359,1			>0.99
**CXCL5**	Mock	3	123.1	264.6	177,6	76,2			
	Mock 7DMA	3	0.0	134.4	44,8	77,6	>0.99		
	USUV Vehicle (D3)	5	120,6	673,0	375.4	208.5		0.95	
	USUV 7DMA (D3)	5	152,2	495,9	259.5	142.3			0.99
	USUV Vehicle (D5)	5	284.7	623.3	412,4	132,0		0.41	
	USUV 7DMA (D5)	4	346.1	803.0	532,9	192,8			>0.99
**CXCL10**	Mock	3	131.3	152.6	142,0	15,1			
	Mock 7DMA	3	65.4	130.3	112,9	24,7	>0.99		
	USUV Vehicle (D3)	5			1389.9	521.6		**0.001**	
	USUV 7DMA (D3)	5			719.4	266.6			**0.004**
	USUV Vehicle (D5)	5	386.2	648.6	519,7	100,8		0.50	
	USUV 7DMA (D5)	5	295.3	934.4	733,2	294,9			>0.99
**CCL11**	Mock	3	65.8	128.7	101,2	32,2			
	Mock 7DMA	2	109.8	222.3	166,1	79,5	>0.99		
	USUV Vehicle (D3)	5	826,8	2059,3	101.7	25.7		>0.99	
	USUV 7DMA (D3)	5	532,4	1164,6	62.5	22.4			0.66
	USUV Vehicle (D5)	5	256.7	634.5	406,1	141,0		>0.99	
	USUV 7DMA (D5)	4	125.6	417.2	218,8	119,4			>0.99

SUPPLEMENTARY TABLE 3

BRAIN

Cytokine	Group	n	Min.	Max.	Mean	SD	p value (Treatment)	p value (Infection)	p value (Inf. x Treat.)
**G-CSF**	Mock	3	0.0	0.0	0.0	0.0			
	Mock 7DMA	3	0.0	0.0	0.0	0.0	>0.99		
	USUV Vehicle (D3)	5	278.0	5337.6	2201.5	1899.0		**<0.0001**	
	USUV 7DMA (D3)	5	102.2	564.9	341.3	209.8			**<0.0001**
	USUV Vehicle (D5)	4	315.1	880.5	562.9	236.2		**<0.0001**	
	USUV 7DMA (D5)	5	154.2	576.7	400.8	165.4			**<0.0001**
**GM-CSF**	Mock	3	0.0	0.0	0.0	0.0			
	Mock 7DMA	3	0.0	0.0	0.0	0.0	>0.99		
	USUV Vehicle (D3)	5	0.0	0.0	0.0	0.0		>0.99	
	USUV 7DMA (D3)	5	0.0	0.0	0.0	0.0			>0.99
	USUV Vehicle (D5)	5	0.0	0.0	0.0	0.0		>0.99	
	USUV 7DMA (D5)	5	0.0	0.0	0.0	0.0			>0.99
**IL-1α**	Mock	3	0.0	0.0	0.0	0.0			
	Mock 7DMA	3	0.0	0.0	0.0	0.0	>0.99		
	USUV Vehicle (D3)	5	0.0	0.0	0.0	0.0		>0.99	
	USUV 7DMA (D3)	5	0.0	0.0	0.0	0.0			>0.99
	USUV Vehicle (D5)	5	0.0	0.0	0.0	0.0		>0.99	
	USUV 7DMA (D5)	5	0.0	0.0	0.0	0.0			>0.99
**IL-1β**	Mock	3	5.4	16.8	7,4	8,6			
	Mock 7DMA	3	5.4	16.0	10,7	5,3	>0.99		
	USUV Vehicle (D3)	5	0	26,3	10.4	11.0		>0.99	
	USUV 7DMA (D3)	5	0	22,8	9.7	9.8			>0.99
	USUV Vehicle (D5)	5	0.0	14.4	5,0	7,0		>0.99	
	USUV 7DMA (D5)	5	0.1	14.5	7,3	5,3			>0.99
**IL-2**	Mock	2	15.3	32.0	15,8	0,7			
	Mock 7DMA	3	5.9	9.0	7,1	1,7	>0.99		
	USUV Vehicle (D3)	5	0	14	8.4	7.4		>0.99	
	USUV 7DMA (D3)	5	8,8	22,6	12.9	5.7			>0.99
	USUV Vehicle (D5)	5	4.3	11.8	7,3	3,7		>0.99	
	USUV 7DMA (D5)	5	9.3	25.1	17,6	5,9			>0.99
**IL-3**	Mock	3	0.0	0.0	0.0	0.0			
	Mock 7DMA	3	0.0	0.0	0.0	0.0	>0.99		
	USUV Vehicle (D3)	5	0.0	0.0	0.0	0.0		>0.99	
	USUV 7DMA (D3)	5	0.0	0.0	0.0	0.0			>0.99
	USUV Vehicle (D5)	5	0.0	0.0	0.0	0.0		>0.99	
	USUV 7DMA (D5)	5	0.0	0.0	0.0	0.0			>0.99
**IL-4**	Mock	3	0.0	0.0	0.0	0.0			
	Mock 7DMA	3	0.0	0.0	0.0	0.0	>0.99		
	USUV Vehicle (D3)	5	0.0	0.0	0.0	0.0		>0.99	
	USUV 7DMA (D3)	5	0.0	0.0	0.0	0.0			>0.99
	USUV Vehicle (D5)	5	0.0	0.0	0.0	0.0		>0.99	
	USUV 7DMA (D5)	5	0.0	0.0	0.0	0.0			>0.99
**IL-5**	Mock	3	0.0	0.0	0.0	0.0			
	Mock 7DMA	3	0.0	0.0	0.0	0.0	>0.99		
	USUV Vehicle (D3)	5	0.0	0.0	0.0	0.0		>0.99	
	USUV 7DMA (D3)	5	0.0	0.0	0.0	0.0			>0.99
	USUV Vehicle (D5)	5	0.0	0.0	0.0	0.0		>0.99	
	USUV 7DMA (D5)	5	0.0	0.0	0.0	0.0			>0.99
**IL-6**	Mock	2	0,0	0,0	0,0	0,0			
	Mock 7DMA	3	7.7	9.4	8,5	0,9	>0.99		
	USUV Vehicle (D3)	5	43,6	377,3	186.0	144.9		>0.99	
	USUV 7DMA (D3)	5	6,9	144,4	62.1	52.8			>0.99

	USUV Vehicle (D5)	5	24.4	48.5	39,6	15,3		>0.99	
	USUV 7DMA (D5)	5	15.3	28.4	20,3	5,3			>0.99
**IL-7**	Mock	1	0,0	7.1	7,1	0,0			
	Mock 7DMA	3	3.3	6.2	5,0	1,5	>0.99		
	USUV Vehicle (D3)	5	3,3	9,9	6.1	3.3		>0.99	
	USUV 7DMA (D3)	5	3,5	6,8	5.5	1.3			>0.99
	USUV Vehicle (D5)	5	4.3	6.8	5,3	1,4		>0.99	
	USUV 7DMA (D5)	5	4.3	6.3	5,2	0,9			>0.99
**IL-9**	Mock	3	162.6	262.1	224,8	54,2			
	Mock 7DMA	3	233.5	340.8	273,6	58,6	>0.99		
	USUV Vehicle (D3)	5	134,1	290,9	214.6	56.9		>0.99	
	USUV 7DMA (D3)	5	216,9	293,8	263.1	40.0			>0.99
	USUV Vehicle (D5)	5	140.4	235.1	191,4	41,4		>0.99	
	USUV 7DMA (D5)	5	199.6	457.2	298,6	98,4			>0.99
**IL-10**	Mock	3	29.7	56.8	44,8	13,8			
	Mock 7DMA	3	32.9	68.7	51,0	17,9	>0.99		
	USUV Vehicle (D3)	5	13,7	50,7	26.9	14.2		>0.99	
	USUV 7DMA (D3)	5	27,5	88	53.9	22.0			>0.99
	USUV Vehicle (D5)	5	26.0	53.4	37,8	13,5		>0.99	
	USUV 7DMA (D5)	5	26.4	84.8	64,0	24,4			>0.99
**IL-12**	Mock	3	0.0	0.0	0,0	0,0			
	Mock 7DMA	3	0.0	42.2	22,1	21,2	>0.99		
	USUV Vehicle (D3)	5	0	14,8	4.8	6.8		>0.99	
	USUV 7DMA (D3)	5	0	31,9	17.1	13.9			>0.99
	USUV Vehicle (D5)	5	0.0	25.9	6,7	11,2		>0.99	
	USUV 7DMA (D5)	5	0.0	38.9	24,0	15,2			>0.99
**IL-12 p70**	Mock	3	0.0	0.0	0.0	0.0			
	Mock 7DMA	3	0.0	0.0	0.0	0.0	>0.99		
	USUV Vehicle (D3)	5	0.0	0.0	0.0	0.0		>0.99	
	USUV 7DMA (D3)	5	0.0	0.0	0.0	0.0			>0.99
	USUV Vehicle (D5)	5	0.0	0.0	0.0	0.0		>0.99	
	USUV 7DMA (D5)	5	0.0	0.0	0.0	0.0			>0.99
**IL-13**	Mock	3	0.0	0.0	0.0	0.0			
	Mock 7DMA	3	0.0	0.0	0.0	0.0	>0.99		
	USUV Vehicle (D3)	5	0.0	0.0	0.0	0.0		>0.99	
	USUV 7DMA (D3)	5	0.0	0.0	0.0	0.0			>0.99
	USUV Vehicle (D5)	5	0.0	0.0	0.0	0.0		>0.99	
	USUV 7DMA (D5)	5	0.0	0.0	0.0	0.0			>0.99
**IL-15**	Mock	3	3.9	46.8	29,9	22,9			
	Mock 7DMA	3	0.0	45.8	30,2	26,2	>0.99		
	USUV Vehicle (D3)	5	0	53,7	15.2	23.6		>0.99	
	USUV 7DMA (D3)	5	0	59,5	26.6	25.8			>0.99
	USUV Vehicle (D5)	5	0.0	37.0	13,9	19,1		>0.99	
	USUV 7DMA (D5)	5	17.6	39.1	32,9	16,1			>0.99
**IL-17**	Mock	3	0.0	0.0	0.0	0.0			
	Mock 7DMA	3	0.0	0.0	0.0	0.0	>0.99		
	USUV Vehicle (D3)	5	0.0	0.0	0.0	0.0		>0.99	
	USUV 7DMA (D3)	5	0.0	0.0	0.0	0.0			>0.99
	USUV Vehicle (D5)	5	0.0	0.0	0.0	0.0		>0.99	
	USUV 7DMA (D5)	5	0.0	0.0	0.0	0.0			>0.99
**IFN-γ**	Mock	3	0,0	0,0	0,0	0,0			
	Mock 7DMA	3	0,0	0,0	0,0	0,0	>0.99		
	USUV Vehicle (D3)	5	0,0	4,8	0.9	2.1		>0.99	
	USUV 7DMA (D3)	5	0,0	6,9	3.7	2.8			>0.99
	USUV Vehicle (D5)	5	0,0	0,0	0,0	0,0		>0.99	
	USUV 7DMA (D5)	5	0,0	0,0	0,0	0,0			>0.99
**LIF**	Mock	3	0.0	0.0	0.0	0.0			

	Mock 7DMA	3	0.0	0.0	0.0	0.0	>0.99		
	USUV Vehicle (D3)	5	0.0	0.0	0.0	0.0		>0.99	
	USUV 7DMA (D3)	5	0.0	0.0	0.0	0.0			>0.99
	USUV Vehicle (D5)	5	0.0	0.0	0.0	0.0		>0.99	
	USUV 7DMA (D5)	5	0.0	0.0	0.0	0.0			>0.99
**CXCL1**	Mock	3	11.2	18.0	15,0	3,5			
	Mock 7DMA	3	16.2	27.2	22,3	5,6	>0.99		
	USUV Vehicle (D3)	5	99,9	553,3	260.6	172.6		>0.99	
	USUV 7DMA (D3)	5	69,2	133,4	105.7	24.4			>0.99
	USUV Vehicle (D5)	5	114.8	229.4	155,8	44,8		**0.016**	
	USUV 7DMA (D5)	5	87.2	177.8	134,2	43,3			0.97
**CXCL5**	Mock	3	0.0	0.0	0.0	0.0			
	Mock 7DMA	3	0.0	0.0	0.0	0.0	>0.99		
	USUV Vehicle (D3)	5	0.0	0.0	0.0	0.0		>0.99	
	USUV 7DMA (D3)	5	0.0	0.0	0.0	0.0			>0.99
	USUV Vehicle (D5)	5	0.0	0.0	0.0	0.0		>0.99	
	USUV 7DMA (D5)	5	0.0	0.0	0.0	0.0			>0.99
**CXCL10**	Mock	3	4.7	21.4	13,2	8,4			
	Mock 7DMA	3	4.2	9.8	7,5	2,9	>0.99		
	USUV Vehicle (D3)	5	60	245,7	143.0	70.3		>0.99	
	USUV 7DMA (D3)	5	64,8	167,5	93.0	42.5			>0.99
	USUV Vehicle (D5)	5	106.2	252.7	144,1	61,3		**0.001**	
	USUV 7DMA (D5)	5	121.6	160.7	144,4	16,2			>0.99
**CCL11**	Mock	3	0.0	12.1	6,1	6,1			
	Mock 7DMA	3	20.1	43.1	28,5	12,7	0.69		
	USUV Vehicle (D3)	5	9,4	117,6	56.8	38.7		>0.99	
	USUV 7DMA (D3)	5	16,7	25,5	21.9	3.3			>0.99
	USUV Vehicle (D5)	5	200.4	464.3	313,7	113,1		**0.0001**	
	USUV 7DMA (D5)	5	137.1	220.7	185,8	32,2			0.094

SUPPLEMENTARY FIGURE 3 – Gating strategy for lymphocytes and meyloid cells in spleen:

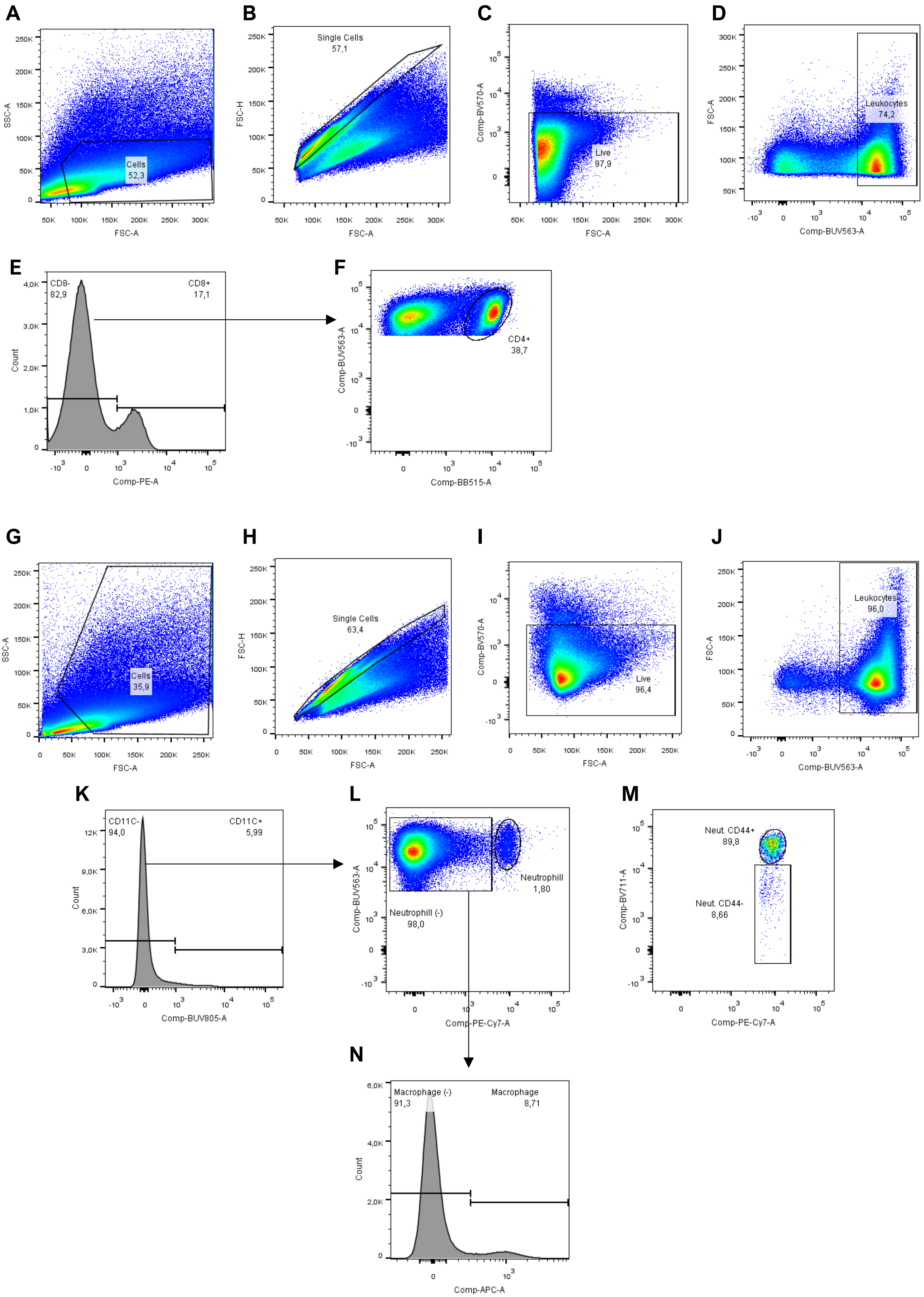

SUPPLEMENTARY FIGURE 4 – Gating strategy for lymphocytes and meyloid cells in blood:

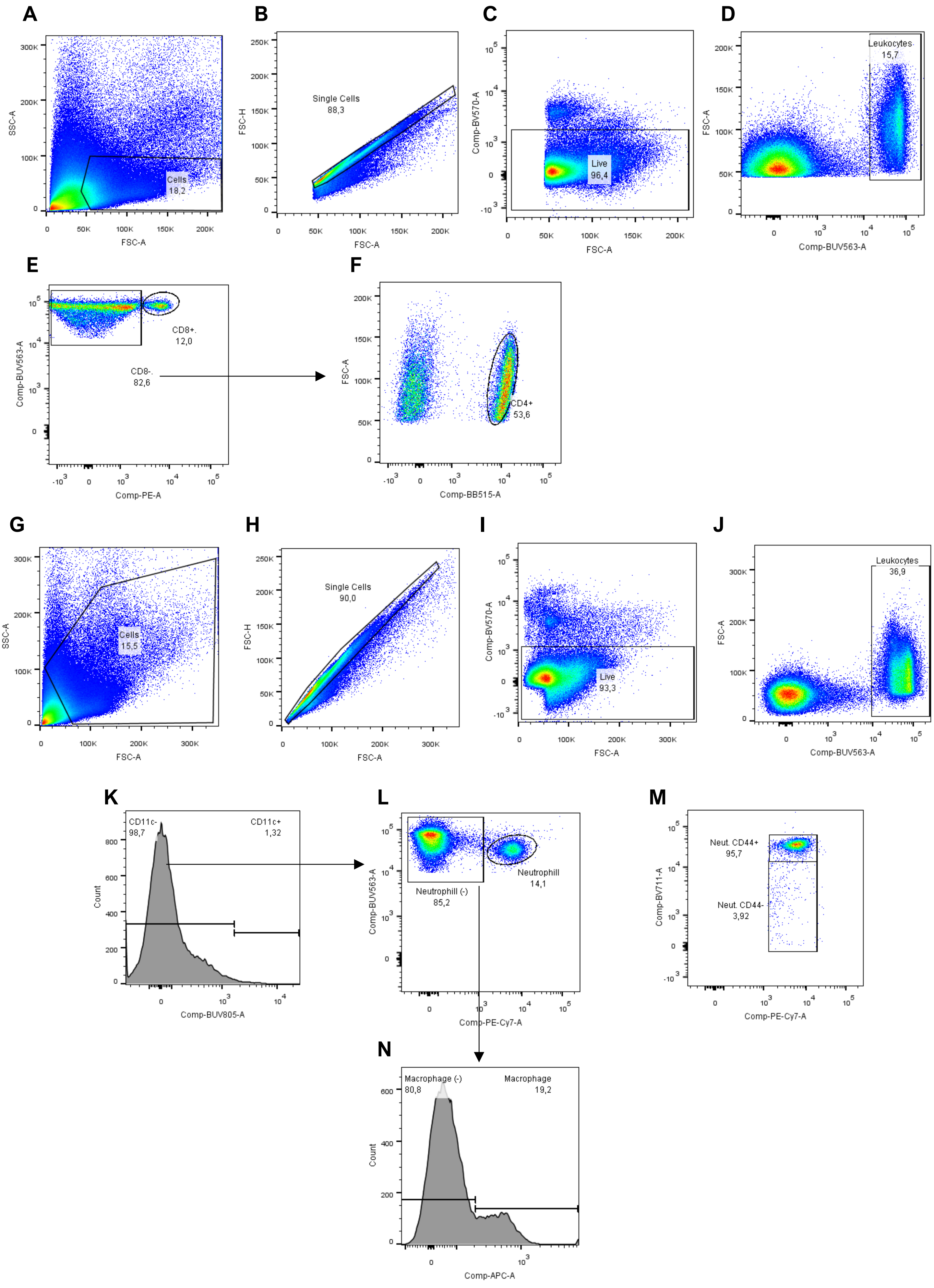

SUPPLEMENTARY FIGURE 5 – Gating strategy for lymphocytes and meyloid cells in brain:

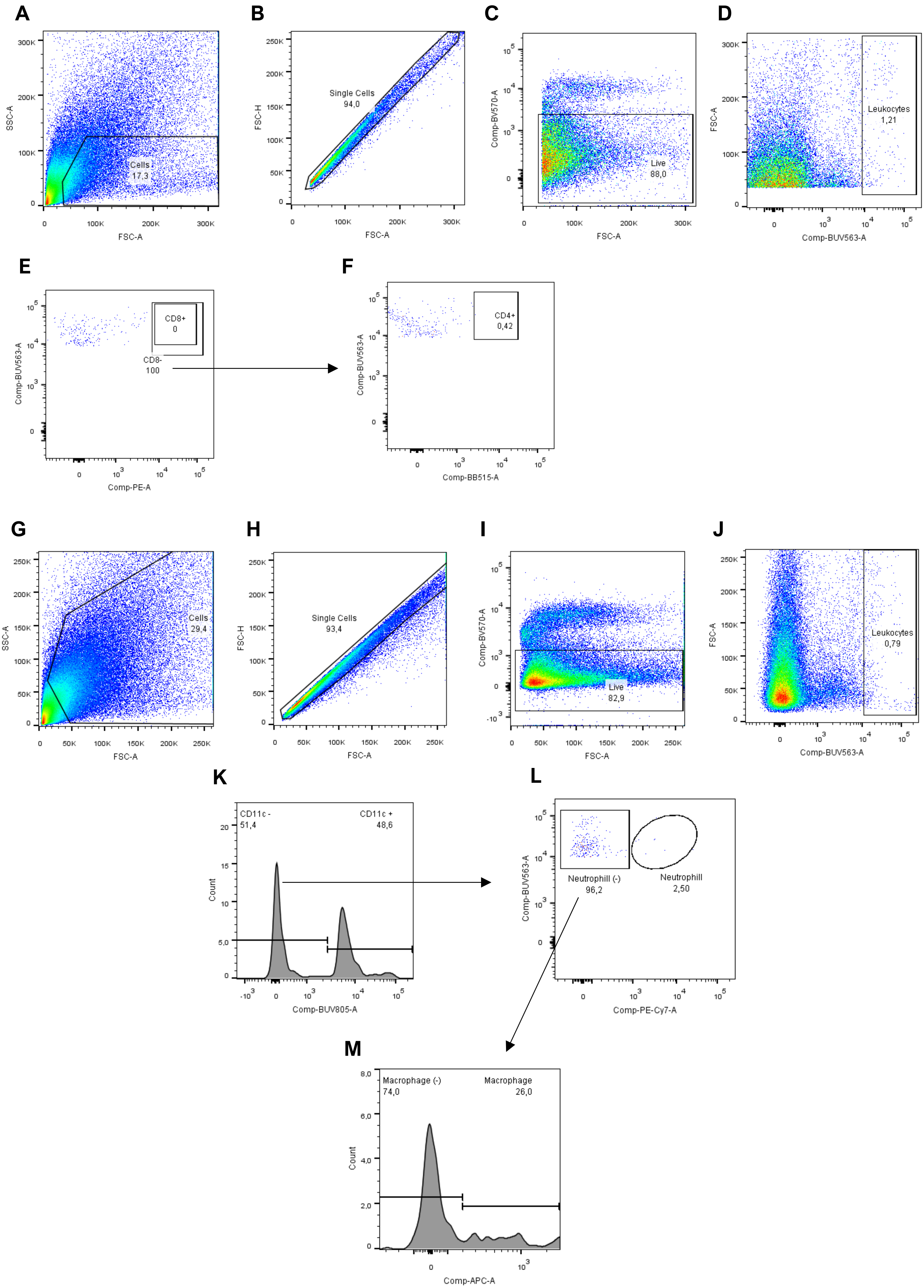

Supplement: Supplementary file 1 [file viruses-17-01639-s001.zip › viruses-3957705-supplementary.pdf]
